# IRAK1 is a therapeutic target that drives breast cancer metastasis and resistance to paclitaxel

**DOI:** 10.1038/ncomms9746

**Published:** 2015-10-27

**Authors:** Zhen Ning Wee, Siti Maryam J. M. Yatim, Vera K Kohlbauer, Min Feng, Jian Yuan Goh, Bao Yi, Puay Leng Lee, Songjing Zhang, Pan Pan Wang, Elgene Lim, Wai Leong Tam, Yu Cai, Henrik J Ditzel, Dave S. B. Hoon, Ern Yu Tan, Qiang Yu

**Affiliations:** 1 Cancer Therapeutics and Stratified Oncology, Genome Institute of Singapore, A*STAR (Agency for Science, Technology and Research), 60 Biopolis Street, 02-01, Biopolis 138672, Singapore; 2 First Affiliated Hospital, Jinan University, Guangzhou 510632, China; 3 Cancer Research Institute, Jinan University, Guangzhou 510632, China; 4 The Kinghorn Cancer Center, Garvan Institute of Medical Research, 384 Victoria Street, Darlinghurst, Sydney, New South Wales 2010, Australia; 5 Cancer Science Institute, National University of Singapore, Singapore 117599, Singapore; 6 School of Pharmacy, Jinan University, Guangzhou 510632, China; 7 Department of Cancer and Inflammation Research, Institute of Molecular Medicine, University of Southern Denmark, Odense 5000, Denmark; 8 Department of Oncology, Odense University Hospital, Odense 5000, Denmark; 9 Department of Molecular Oncology, John Wayne Cancer Institute, Santa Monica, California 90404, USA; 10 Department of General Surgery, Tan Tock Seng Hospital, Singapore 308433, Singapore; 11 Department of Physiology, Yong Loo Lin School of Medicine, National University of Singapore, Singapore 117597, Singapore; 12 Cancer and Stem Cell Biology, DUKE-NUS Graduate Medical School of Singapore, Singapore 169857, Singapore

## Abstract

Metastatic tumour recurrence due to failed treatments remains a major challenge of breast cancer clinical management. Here we report that interleukin-1 receptor-associated kinase 1 (IRAK1) is overexpressed in a subset of breast cancers, in particular triple-negative breast cancer (TNBC), where it acts to drive aggressive growth, metastasis and acquired resistance to paclitaxel treatment. We show that IRAK1 overexpression confers TNBC growth advantage through NF-κB-related cytokine secretion and metastatic TNBC cells exhibit gain of IRAK1 dependency, resulting in high susceptibility to genetic and pharmacologic inhibition of IRAK1. Importantly, paclitaxel treatment induces strong IRAK1 phosphorylation, an increase in inflammatory cytokine expression, enrichment of cancer stem cells and acquired resistance to paclitaxel treatment. Pharmacologic inhibition of IRAK1 is able to reverse paclitaxel resistance by triggering massive apoptosis at least in part through inhibiting p38-MCL1 pro-survival pathway. Our study thus demonstrates IRAK1 as a promising therapeutic target for TNBC metastasis and paclitaxel resistance.

## Abstract (web summary)

Triple negative breast cancer (TNBC) patients often acquire resistant to chemotherapy. In this study, the authors identify the IRAK1 as the crucial driver of NF-κB-related cytokine secretion involved in TNBC metastasis and therapy resistance.

## Graphical abstract

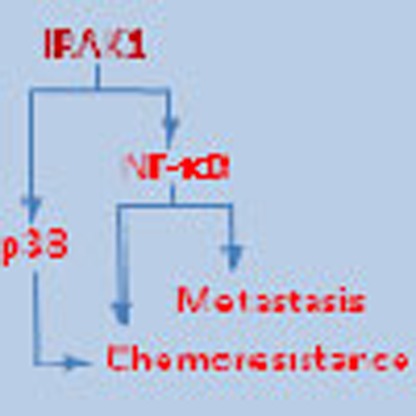

Triple-negative breast cancer (TNBC) accounts for ∼15–20% of all breast cancers^1^ and is frequently associated with an overall poor prognosis characterized by a higher rate of recurrence and distant metastasis. Although chemotherapy is effective initially in a subset of patients, the disease often recurs and progresses aggressively due to acquired chemoresistance, resulting in a shorter overall survival as compared with other subtypes of breast cancer^2^. Despite being a major cause of mortality, treatment options for advanced TNBC remains limited, necessitating identification of new therapeutic strategies that target metastatic recurrence and chemoresistance.

Inflammatory response plays a crucial role in cancer progression^3^,^4^,^5^. In particular, inflammatory cytokine and chemokine production, elicited by pathways such as nuclear factor-κB (NF-κB), Jak/Stats and interferons, have been linked to cancer initiation, metastasis and chemoresistance^6^,^7^,^8^. In breast cancer, constitutive activation of NF-κB has been found to be more frequent in TNBC, which can be elicited by both autocrine and paracrine mechanisms, leading to expression of a myriad of downstream targets including inflammatory cytokines, such as interleukin (IL)-6, IL-8, CXCLs and anti-apoptotic genes to confer aggressive growth, stemness and chemoresistance^9^,^10^,^11^,^12^. Although NF-κB appears to be an excellent target for cancer therapy, development of NF-κB inhibitors have failed to provide clinical benefits due to severe toxicity seen in normal cells^13^,^14^,^15^,^16^. As such, efforts have been invested to develop therapeutic strategies that selectively target cancer-specific NF-κB downstream events, to spare the normal cells^17^. Alternatively, we envision that exploration of actionable upstream events that confers NF-κB dependency in cancer cells but not in normal cells may also warrant therapeutic opportunities for treating NF-κB-driven human cancers such as TNBC.

Toll-like receptors (TLRs) and IL-1 receptor (IL-1R) signalling engages IL-1R-associated kinase IRAK1 and IRAK1 phosphorylation, to drive downstream events including NF-κB and interferon signalling in inflammatory responses, whereby these events have been recently implicated in tumorigenesis^18^,^19^,^20^,^21^. In recent times, it has been shown that pharmacologic inhibition of IRAK1/4 is efficacious in targeting myelodysplastic syndromes and acute lymphoblastic leukemia^18^,^20^.

In this present study, we report an oncogenic role of IRAK1 in TNBC metastasis, recurrence and acquired resistance to paclitaxel through both NF-κB-dependent and -independent mechanisms. Importantly, we show that pharmacologic inhibitors of IRAK1, including a natural product, are robustly active against TNBC progression and are able to tackle paclitaxel resistance, thus providing a readily explorable therapeutic strategy for targeting refractory metastatic TNBC, which is currently incurable.

## Results

### *IRAK1* is overexpressed in a subset of breast cancers

**Figure 1:**
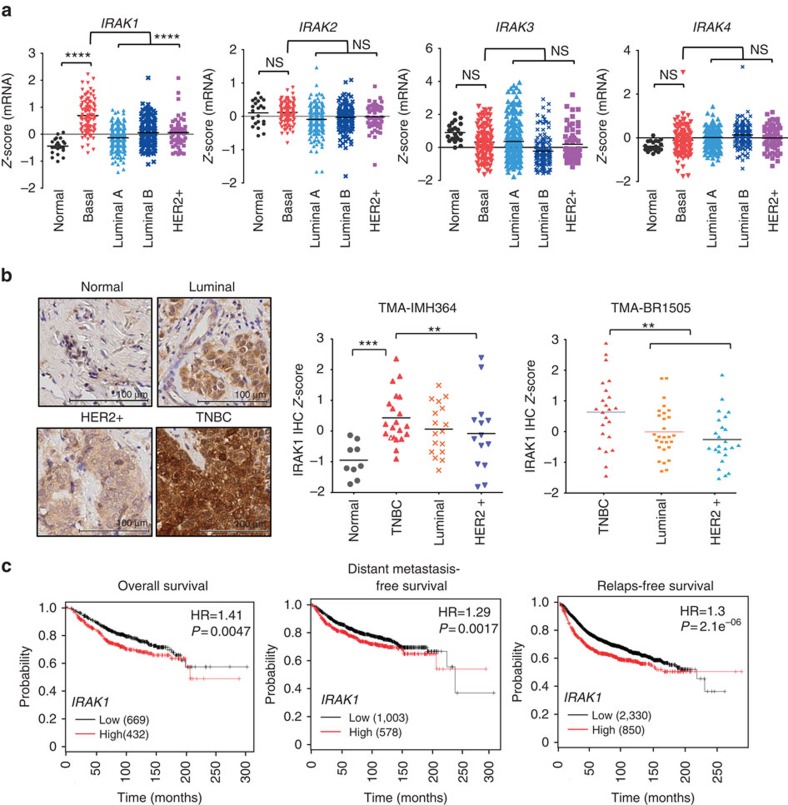
*IRAK1* overexpression in breast cancers. (**a**) The Cancer Genome Altas analysis shows the expression levels of *IRAK* family members across different subtypes of breast cancers and normal tissues. Normal, *n*=22; basal-like, *n*=98; luminal A, *n*=232; luminal B, *n*=129; HER2, *n*=58. (**b**) TMA analysis of IRAK1 protein expression in different subtypes of breast cancers. Shown are representative IHC images of IRAK1 expression (left) and quantifications in two different TMA cohorts as indicated (right). (**c**) Kaplan–Meier analyses of relapse-free survival, overall survival and distant metastasis-free survival of breast cancer patients from the KM plotter database (www.kmplot.com)^50^. *P*-values were calculated with log-rank (Mantel–Cox) test. Patients were stratified into ‘low' and ‘high' *IRAK1* expression based on autoselect best cutoff. **P<0.01, ****P<0.0001, NS, not significant; Tukey's multiple comparisons test.

In search of the upstream molecular events of NF-κB signalling that might be aberrantly expressed in breast cancers, we interrogated The Cancer Genome Altas database and found that *IRAK1*, a known upstream regulator of NF-κB, is significantly overexpressed in various subtypes of breast tumours in comparison with normal breast epithelium (Fig. 1a, *P*<0.0001, Tukey's multiple comparisons test), whereas the other three members of the *IRAK* family *IRAK2-4* did not show such a difference (Fig. 1a). Of further note, the expression levels of *IRAK1* were in particular higher among tumours of the basal subtype when compared with other subtypes (Fig. 1a, *P*<0.0001, Tukey's multiple comparisons test).

Immunohistochemistry (IHC) analyses of two commercial tissue microarrays (TMAs) (IMH364 and BR1505), which comprises two independent cohorts of breast tumour specimens with different subtypes confirmed the upregulation of IRAK1 protein expression in breast cancers, in particular among TNBCs (Fig. 1b, *P*<0.01, Tukey's multiple comparisons test).

To explore a role of *IRAK1* in clinical outcomes, we performed meta-analyses using Kaplan–Meier plotter online breast cancer survival analysis (www.kmplot.com). The results revealed that high *IRAK1* expression positively correlated with reduced overall survival, distant metastasis-free survival and relapse-free survival ((*P*=0.0047, *P*=0.017 and *P*=2.1 × 10^6^, respectively, Fig. 1c), suggesting a prognostic value of *IRAK1* in breast cancers. These findings suggest a potential role of *IRAK1* in breast tumorigenesis.

### Inhibition of *IRAK1* abrogates aggressive growth of TNBC

**Figure 2:**
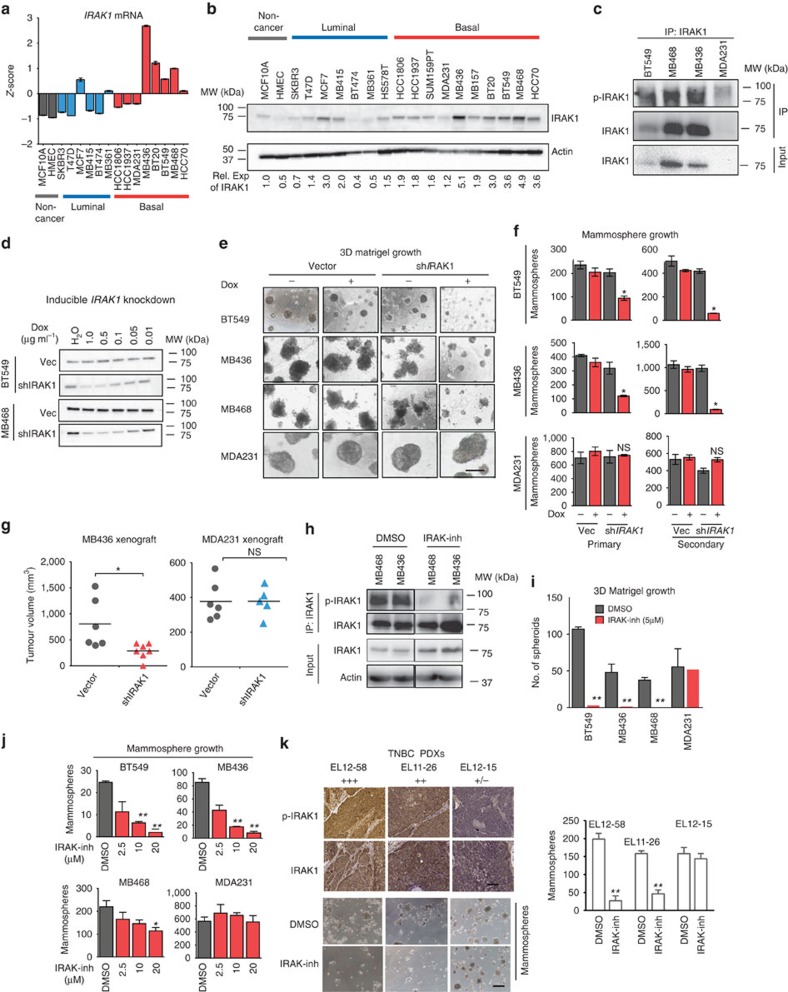
*IRAK1* knockdown and pharmacologic inhibition impair the aggressive growth phenotypes of TNBC cells. (**a**) Qantitative reverse transcriptase–PCR analysis of *IRAK1* expression in a panel of breast cancer cell lines. (**b**) Western blot analysis of IRAK1 expression. Below shows the densitometric quantification of IRAK1 expression relative to MCF10A. (**c**) Western blot analysis of immnoprecipiated IRAK1 for phosphorylation (T209) in indicated TNBC cell lines. (**d**) Western blotting showing the knockdown efficiency of inducible shIRAK1 or nonspecific shRNA vector control treated with different concentrations of Dox. (**e**) Representative images of 3D Matrigel growth of indicated TNBC cells treated with or without Dox (0.5 μg ml^−1^) for 7 days. Scale bars, 100 μm. (**f**) Bar graphs showing the quantifications of primary and secondary mammosphere formation in TNBC cells treated with Dox (0.5 μg ml^−1^), to induce IRAK1 knockdown. (**g**) Scatter plot showing the MB436 and MDA231 xenograft tumour growth for 36 days in female NOD/SCID mice carrying cells' vector control (*n*=6) or inducible shIRAK1 (*n*=6), and treated with Dox (100 mg kg^−1^) for 21 days. (**h**) Western blotting showing the effects of IRAK-inh (5 μM) on p-IRAK1 (T209) in immunoprecipitated total IRAK1. (**i**,**j**) Effects of IRAK-inh on 3D Matrigel growth and mammosphere formation. (**k**) Effects of IRAK1-inh (5 μM) on PDX-derived mammosphere formation. IRAK1 and p-IRAK1 (S376) IHC staining (top); mammosphere growth (bottom). Error bars represent s.e.m, *n*=3. *P<0.05 compared with the vector control or DMSO. NS, not significant. *P*-values were calculated with two-tailed *t*-test.

To examine whether the above finding in breast cancer clinical samples can be similarly found in breast cancer cell lines *in vitro*, we analysed a panel of 14 breast cancer cell lines including luminal, basal and two non-cancerous breast epithelial MCF10A and HMEC lines. Reverse transcriptase–PCR analysis shows that *IRAK1* messenger RNA is upregulated in over 80% of breast cancer cell lines as compared with MCF10A and HMEC, with *IRAK1* expression being markedly higher in basal lines compared with luminal lines (Fig. 2a). In contrast, *IRAK4*, the other IRAK family member with kinase activity, did not show a consistent difference between luminal and basal lines (Supplementary Fig. 1A). A similar result was also obtained in GOBO database analysis that contains expression data of 55 breast cancer cell lines (http://co.bmc.lu.se/gobo/) (Supplementary Fig. 1B). Western blot analysis confirmed the reverse transcriptase–PCR results showing higher levels of IRAK1 protein in basal compared with luminal lines (Fig. 2b). We further showed that the three basal/TNBC lines BT549, MDA-MB436 (thereafter named MB436) and MDA-MB468 (thereafter named MB468) with higher levels of IRAK1 also expressed high levels of phosphorylated IRAK1 compared with IRAK1-low-expressing MDA-MB-231 cells (thereafter named MDA231 cells), therefore indicating the active state of IRAK1 in these cells (Fig. 1c).

To study a functional role of *IRAK1* in TNBC, we knocked down *IRAK1* expression with two independent small hairpin RNA (shRNA) constructs that express green fluorescence protein (GFP) in TNBC cell lines. *IRAK1* knockdown in *IRAK1*-high-expressing BT549 and MB436 cells caused a modest effect on cell proliferation by day 7, but prolonged culture till day 20 led to gradual depletions of GFP-positive cells, but this was not seen in *IRAK1*-low-expressing MDA231 cells (Supplementary Fig. 1C–E). This suggests that IRAK1 has a role in conferring survival advantage in TNBC cells, although not robustly seen in the monolayer culture condition.

We next sought to determine the effect of IRAK1 knockdown on aggressive growth properties of TNBC, such as three-dimensional cultures associated with invasive growth and cancer-initiating cells or cancer stem cells (CSCs). To facilitate the study, we made use of a doxycycline (Dox)-inducible knockdown system in which adding Dox in the tissue culture resulted in depletion of IRAK1 (Fig. 2d). Dox-induced *IRAK1* knockdown resulted in robust inhibitions of three-dimensional (3D) Matrigel growth in *IRAK1*-high-expressing cells (BT549, MB436 and MB-468) but not in MDA231 cells that express a low level of *IRAK1* (Fig. 2e). Moreover, IL-1β-evoked Matrigel cell invasion in MB436 and BT549 cells was also markedly impaired upon *IRAK1* knockdown, whereas MDA231 cells did not respond to IL-1β treatment and thus was insensitive to *IRAK1* knockdown (Supplementary Fig. 1F).

To study a role of IRAK1 in CSCs, we assess the ability of TNBC cells to form mammospheres in serum-free suspension culture as a functional assay for CSCs^22^, as there is no single common marker that can be reliably used to assess the cancer stemness across these different TNBC cell lines. Similarly, Dox-induced *IRAK1* knockdown impaired both the primary and secondary mammosphere formation in *IRAK1* high-expressing TNBC cell lines, but not MDA231 cells, indicating a role of *IRAK1* in both tumour-initiating and self-renewal capacity (Fig. 2f and Supplementary Fig. 1G).

These *in vitro* effects were also seen *in vivo* as MB436, but not MDA231 xenograft tumours bearing *IRAK1* shRNA exhibited significant growth inhibition in Dox-treated non-obese diabetic/severe combined immunodeficiency (NOD/SCID) mice for 21 days (Fig. 2g). These results demonstrated an indispensible role of *IRAK1* in both the aggressive growth of TNBC cells *in vitro* and the tumorigenicity *in vivo*.

We further asked whether pharmacologic inhibition of IRAK1 recapitulates the *IRAK1* gene knockdown phenotypes. IRAK-inh is a commercially available IRAK1/4 inhibitor, which has recently been shown to have potent activity against IRAK1 in myelodysplastic syndromes^18^. To this end, we examined the sensitivity of a panel of 14 breast cancer cell lines including luminal, basal and non-cancerous mammary epithelial cell lines MCF10A and HMEC to IRAK-inh. The results show a significant positive correlation between the levels of *IRAK1* expression and IRAK-inh sensitivity (Pearson's *R* score=0.89, *P*<0.0001; Supplementary Fig. 2A). Similar to *IRAK1* knockdown, IRAK-inh treatment was more effective against TNBC cells that express high levels of *IRAK1*, such as BT549, MB436 and MB468, but not in MDA231 and SUM159PT that express low levels of IRAK1 (Supplementary Fig. 2A). It also had little effect on MCF10A and HMEC cells, as well as most of the luminal cell lines, indicating the potential cancer selectivity (Supplementary Fig. 2A). As expected, IRAK-inh treatment effectively abolished p-IRAK1 in TNBC cells (Fig. 2h), leading to marked inhibition of 3D Matrigel and mammosphere growth in BT549, MB436 and MB468 cells, but neither in MDA231 and SUM159PT cells nor in MCF10A and HMLE cells (Fig. 2i,j and Supplementary Fig. 2C,D). Lack of sensitivity of MDA231 cells to IRAK-inh may indicate a deficiency of IL-1/IRAK1 signalling in these cells. Indeed, treatment with recombinant IL-1β induced a fast induction of p-IRAK1 in IRAK-inh-sensitive MB436 cells, which could be abrogated by IRAK-inh treatment, whereas the same treatment did not elicit a similar response in MDA231 cells (Supplementary Fig. 2E).

Lastly, we examined the effects of IRAK1-inh on the *in-vitro* mammosphere formation of three patient-derived xenograft (PDX) tumours^23^ that express different levels of IRAK1 (Fig. 2k). As expected, IRAK-inh treatment resulted in robust inhibition of mammosphere growth of the two PDXs that express higher levels of IRAK1 (EL12–58 and EL11–26) but not the PDX that expresses a lower level of IRAK1 (EL12–15) (Fig. 2k). Together, these data, based on *in vitro*, *in vivo* and patient-derived samples, strongly suggest that small-molecule inhibitor of IRAK1 is active against the aggressive growth of TNBCs that overexpress *IRAK1*.

### IRAK1-mediated cytokine secretion drives tumorsphere growth

**Figure 3:**
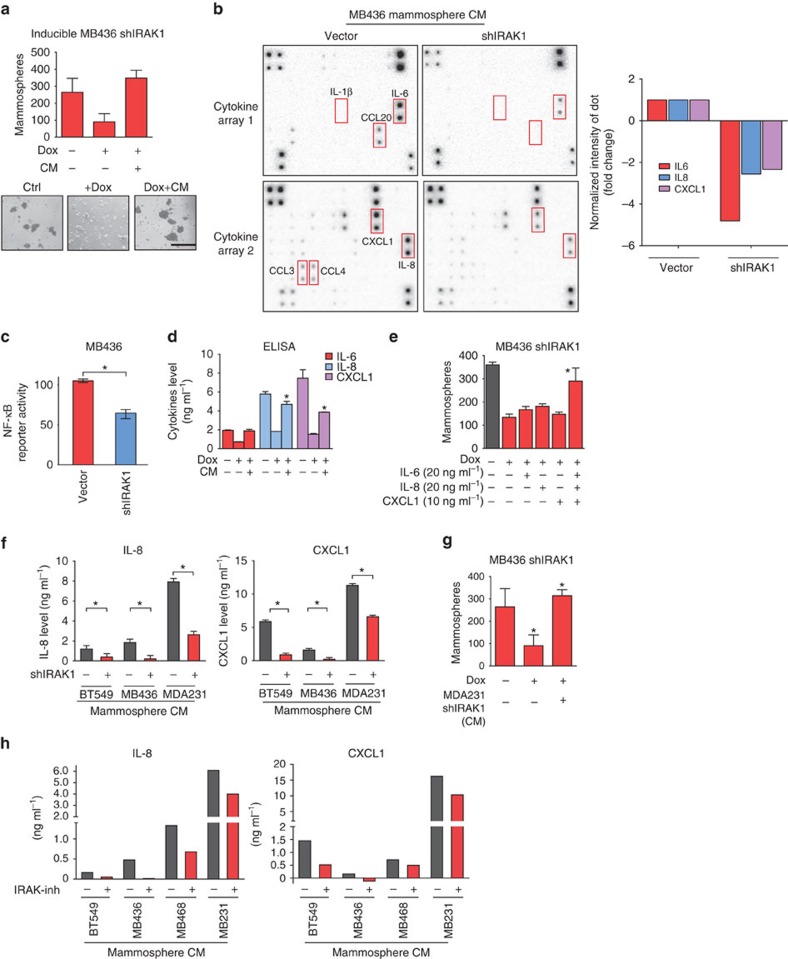
IRAK1-dependent cytokine secretions are required for mammosphere formation. (**a**) Mammosphere formation of MB436 shIRAK1 cells treated with and without Dox (0.5 μg ml^−1^) for IRAK1 knockdown. CM, conditioned medium from mock-treated MB436 shIRAK1 cells. Quantifications (top) and representative images (bottom). Scale bars, 100 μm. (**b**) Cytokine Ab array profiling of cytokine secretions in the growth medium of MB436 cells expressing vector control or shIRAK1 in the presence of Dox (left). Quantifications of IL-6 and IL-8 and CXCL1 changes in shIRAK1 cells relative to the vector control cells (right). (**c**) NF-κB luciferase reporter activity in indicated MB436 cells in the presence of Dox. (**d**) ELISA assay showing the secretion of IL-6, IL-8 and CXCL1 in MB436 shIRAK1 cells with or without Dox or conditional medium from mock-treated cells. (**e**) Mammosphere formation assay in the presence of Dox or recombinant cytokines individually or in combination as indicated. (**f**) ELISA assay showing the secretion of IL-8 and CXCL1 in indicated TNBC cells expressing vector control or shIRAK1. (**g**) Mammosphere formation of MB436 shIRAK1 cells treated with Dox alone or together with CM from MDA231 shIRAK1 cells. (**h**) Effects of IRAK-inh (5 μM) treatment on cytokine secretions of indicated TNBC cells. Error bars represent s.e.m, *n*=3. *P<0.05, **P<0.01. *P*-values were calculated with two-tailed *t* test.

Given that previous studies have shown roles of NF-κB-dependent cytokine production in supporting breast cancer CSCs^12^,^24^,^25^, we reasoned that the reduced mammosphere growth following *IRAK1* knockdown might result from reduced cytokine production and thus should be rescued by conditional medium of untreated cells. Indeed, adding back the supernatant of control MB436 sphere cells to the *IRAK1*-depleted MB436 cells restored the sphere-forming capacity (Fig. 3a) and suggested that IRAK1-regulated cytokine production might be crucial to TNBC tumour growth through induction of the CSC subpopulation.

To identify the cytokines that are crucial for IRAK1-regulated CSC growth, we used RayBio human cytokine Ab array and performed quantitative cytokine profiling of conditional sphere growth medium of MB436 cells with and without *IRAK1* knockdown. We identified a number of cytokines that showed reduced secretion on *IRAK1* knockdown. Among them, IL-6, IL-8 and CXCL1, previously shown to be important for breast cancer CSCs^10^,^11^,^26^, were the top three cytokines that seemed to be most abundantly detected and downregulated following *IRAK1* knockdown (Fig. 3b). Consistently, knockdown of *IRAK1* caused significant inhibition of NF-κB reporter activity (Fig. 3c).

Enzyme-linked immune-sorbent assay (ELISA) confirmed that *IRAK1* knockdown reduced the secretions of IL-6, IL-8 and CXCL1 in the supernatant of MB436 cells, which was restored on replacement with medium of mock-treated cells (Fig. 3d). We further showed that addition the pooled three recombinant cytokines was able to fully restore the mammosphere growth of IRAK1-depleted cells, although the individual addition of these cytokines was insufficient to achieve the same (Fig. 3e). These findings identify collective roles of IRAK1-regulated cytokines in maintaining the CSC-related mammophere formation.

We further showed that *IRAK1* repression strongly reduced cytokine secretions in BT549 and MB436 but to a much lesser extent in MDA231 (Fig. 3f). We postulated that the remaining levels of cytokines in MDA231 cells after IRAK1 knockdown is still sufficient to promote mammosphere formation. Indeed, the conditioned medium harvested from MDA231 shIRAK1 cells was sufficient to fully rescue the inhibition on the mammosphere formation of MB436 shIRAK1 cells (Fig. 3g). Thus, unlike other TNBC cells with high levels of *IRAK1*, the NF-κB-related cytokine productions in MDA231 cells is substantially less dependent on *IRAK1*, which might account for the lack of sensitivity of MDA231 cells to *IRAK1* depletion. Consistent with the above phenotypes, we saw that IRAK-inh treatment resulted in a similar strong reduction of these cytokine secretions in IRAK-inh-sensitive TNBC cells but to a much lesser extent in IRAK1-inh-resistant MDA231 cells (Fig. 3h).

### TNBC towards metastasis exhibits gain of IRAK1 dependency

**Figure 4:**
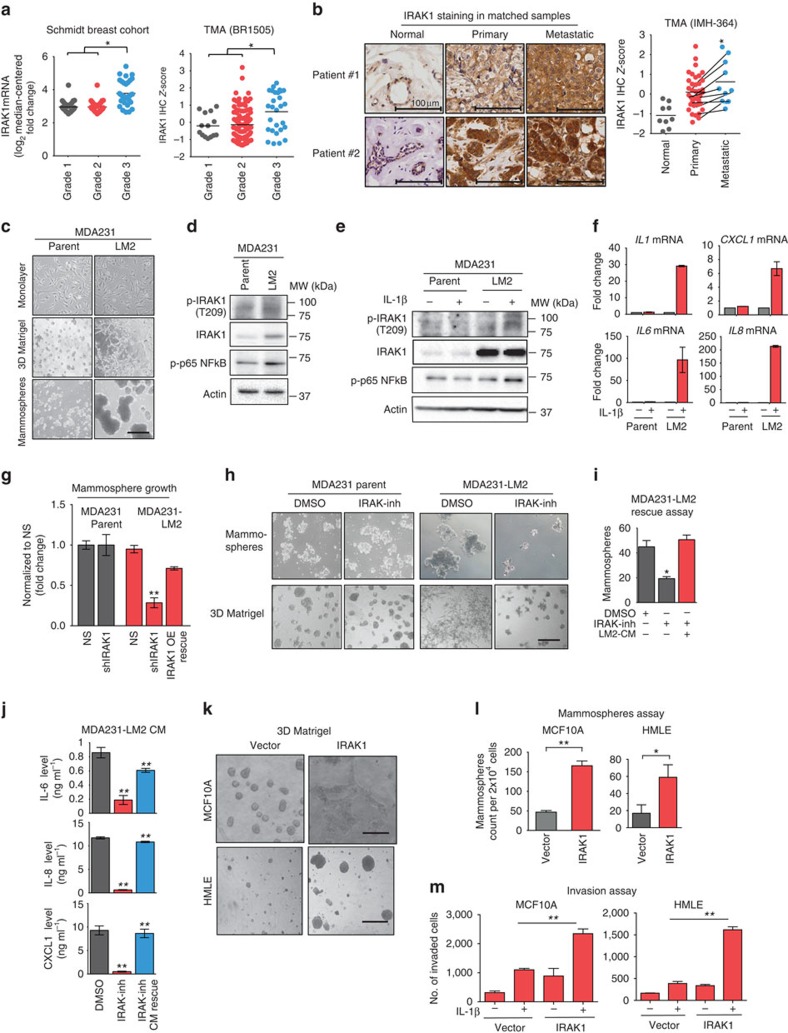
TNBC metastasis shows increased IRAK1 expression/activity and gain of IRAK1 dependency. (**a**) Scatter plots showing the *IRAK1* mRNA levels in Schmidt Breast Cohort (GSE11121) composed of 200 breast cancer tumours of different clinical grades (Grade 1, *n*=29; Grade 2, *n*=136; Grade 3, *n*=35) (left), and IRAK1 IHC staining results in TMA-BR1505 with 150 breast tumour cores of different clinical grades (Grade 1, *n*=14; Grade 2, *n*=104; Grade 3, *n*=28) (right). (**b**) Representative IHC images of IRAK1 protein expression in matched normal, primary and metastatic tumours from two patients (left). Scale bars, 100 μm. Scatter plot showing IRAK1 protein expression in TMA-IMH364, including nine paired primary and matched metastatic tumours, and indicated by the black lines (right). (**c**) Phase-contrast microscopic images of MDA231 parental and MDA231-LM2 cells cultured in monolayer, mammospheres and 3D Matrigel conditions. Scale bars, 100 μm. (**d**) Western blotting showing the expression of indicated proteins. (**e**) Western blotting showing the expression of indicated proteins in the absence or presence of IL-1β (10 ng ml^−1^) for 24 h. (**f**) Quantitative PCR analysis showing the relative expression of indicated cytokines in cells treated in **e**. (**g**) Mammosphere formation assay of MDA231 and MDA231-LM2 cells expressing vector, shIRAK1, or together with ectopic IRAK1 for rescue. (**h**) Representative phase-contrast images in mammosphere and 3D Matrigel gel, treated with or without IRAK-inh (5μM) for 7 days. Scale bars, 100 μm. (**i**) Mammosphere growth of MDA231-LM2 cells treated with 5 μM IRAK-inh, with and without adding conditioned medium (CM) from mock-treated MDA231-LM2 cells. (**j**) ELISA quantification of indicated cytokine levels in **i**. (**k**) 3D Matrigel growth of MCF10A and HMLE cells expressing vector control or ecotopic IRAK1. Scale bars, 100 μm. (**l**) Mammosphere assay of cells in **k**. (**m**) Invasion assay of cells in **k** treated with recombinant IL-1b (10 ng ml^−1^) for 3 days. Error bars represent s.e.m. *n*=3. *P<0.05, **P<0.01. *P*-values were calculated with two-tailed *t*-test.

Given a strong role of IRAK1 in invasive growth and mammosphere formation, we next evaluated the clinical relevance of *IRAK1* expression in relation to breast cancer progression. We found that *IRAK1* is expressed in much higher levels in poorly differentiated high grade (grade 3) tumours compared with relatively well-differentiated grade 1–2 tumours, as revealed by both Oncomine analysis and IHC verification (Fig. 4a). Moreover, IRAK1 expression shows progressive increase from normal adjacent breast tissues to matched primary and node metastasis tissues as shown in six out of nine primary metastasis pairs in the TMA IMH-364 (Fig. 4b), indicating an association of IRAK1 expression with metastatic progression. Consistent with this, Oncomine analysis of two independent breast cancer cohorts showed a significant positive correlation of *IRAK1* expression with metastasis events at 5 years post surgery (Supplementary Fig. 3A).

To recapitulate the above clinical observation *in vitro*, we compared the MDA231 and its derived lung metastatic subline MDA231-LM2 (ref. 27). Although the two lines exhibited no apparent difference in monolayer morphology, metastatic MDA231-LM2 cells compared with MDA231 cells displayed much more aggressive phenotypes when cultured in 3D Matrigel and mammosphere growth condition (Fig. 4c). Consistent with the aggressive growth properties, MDA231-LM2 cells showed increased levels of both total IRAK1 and p-IRAK1, as well as p-p65NF-κB compared with the parental MDA231 cells (Fig. 4d). They also exhibited increased transcription and secretion of IL-6, IL-8 and CXCL1 (Supplementary Fig. 3B,C). Of note, *ILIA* and *IL1B*, as well as *IL1R*, which encodes IL-1R, also showed increased expression in MDA231-LM2 cells (Supplementary Fig. 3B), suggesting an augmented autocrine feedback loop activating IL-1/IRAK1 signalling.

Moreover, compared with parental MDA231 cells that were unresponsive to IL-1β treatment, MDA231-LM2 cells now became responsive to recombinant IL-1β treatment by showing robust p-IRAK1 and cytokine inductions (Fig. 4e,f). These results indicated a gain of IL-1/IRAK1 signalling activity in metastatic MDA231-LM2 cells.

Accordingly, an *IRAK1* shRNA targeting the 3′-untranslated region (UTR) of *IRAK1* was able to effectively inhibit the 3D Matrigel growth and mammosphere growth of MDA231-LM2 cells, whereby this inhibition was readily rescued by ectopic IRAK1 (Fig. 4g and Supplementary Fig. 3D). Consistently, IRAK-inh phenocopied the effects of *IRAK1* knockdown, leading to impaired 3D Matrigel and mammosphere growth in MDA231-LM2 but in its parental line (Fig. 4h). Again, application of cell culture supernatant of mock-treated MDA231-LM2 cells to the culture of IRAK1-inh-treated MDA231-LM2 cells led to a complete rescue of mammosphere growth (Fig. 4i), which echoed the restored levels of cytokines (Fig. 4j).

Importantly, unlike *IRAK1* knockdown that selectively affects MDA231-LM2 cells but not MDA231 cells, directly targeting NF-κB by knocking down of p65 NF-κB/RelA subunit was able to eliminate the growth of both MDA231 and MDA231-LM2 cells (Supplementary Fig. 3E,F). This indicates that although NF-κB is important for both parental and metastatic MDA231, it has gained preferential dependency on IRAK1 in MDA231-LM2 cells, resulting in selective susceptibility to IRAK1 interference.

Moreover, as both the kinase function and scaffold function have been implicated in IRAK1 signalling in a context-dependent manner^28^, we sought to determine whether or not the kinase activity of IRAK1 is required for the aggressive growth of MDA231-LM2 cells. As shown in Supplementary Fig. 4A, expression of ectopic wild-type IRAK1 in MDA231-LM2 cells had no obvious effect on the monolayer growth but enhanced the aggressive growth phenotypes. In contrast, ectopic expression of a kinase-dead IRAK1 that carries a point mutation in the ATP-binding pocket (K239S) induced strong inhibitory effects on 3D Matrigel and mammosphere growth, although having no effect on monolayer cell growth (Supplementary Fig. 4A). Consistently, wild-type IRAK1 increased the secretions of IL-6, IL-8 and CXCL1, whereas IRAK1 K239S mutant yielded opposite effects (Supplementary Fig. 4B), showing a dominate negative effect of IRAK1 kinase mutant. These results confirmed an indispensible role of IRAK1 kinase activity in aggressive phenotypes of TNBC.

Lastly, we asked whether IRAK1 is functionally sufficient to enable aggressive growth. We explored the effects of ecotopic IRAK1 expression on non-cancerous mammary epithelial cells MCF10A and HMLE. Although ecotopic *IRAK1* expression in MCF10A cells only had a modest effect on cell proliferation cultured on monolayer (Supplementary Fig. 5A), it remarkably enhanced soft agar growth (Supplementary Fig. 5B), indicating its ability for oncogenic transformation. Ectopic *IRAK1* also enhanced the 3D Matrigel and mammosphere growth in MCF10A and HMLE cells (Fig. 4k,l), and induced the expressions of *IL6*, *IL8* and *CXCL1* mRNAs (Supplementary Fig. 5C). Moreover, expression of ectopic *IRAK1* greatly potentiated IL-1β-induced invasion in MCF10A and HMLE cells (Fig. 4m). Taken together, these findings indicate that IRAK1 is functionally sufficient to enable malignant transformation and aggressive growth of mammalian epithelial cells.

### Inhibiting IRAK1 impairs TNBC growth and metastasis *in vivo*

**Figure 5:**
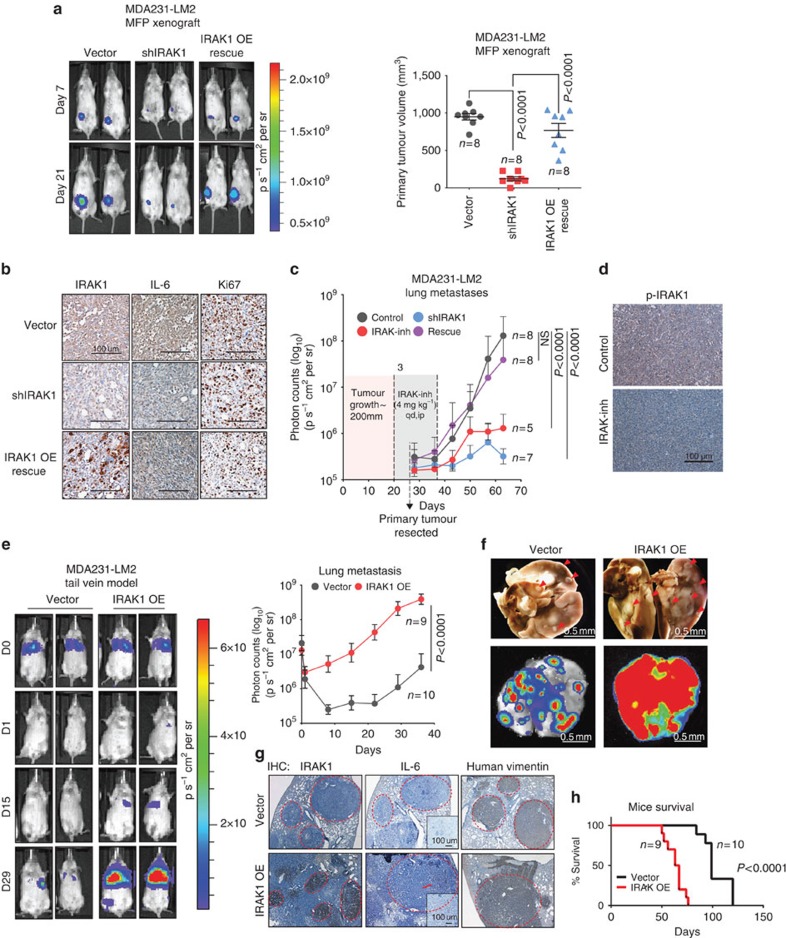
IRAK1 is both required and sufficient to promote TNBC growth and metastasis *in vivo*. (**a**) Representative bioluminescent imaging (BLI) showing the mammary fat pad (MFP) xenograft tumour growth of indicated MDA231-LM2 cells in NOD/SCID mice (left). Scatter plot showing the tumour volumes at day 27. (**b**) Representative IHC staining of indicated proteins on xenograft tumours harvested in **a**. (**c**) BLI curves showing the development of lung metastasis of MDA231-LM2 cells in **a**, or treated with IRAK-inh (4 mg kg^−1^, qd, intraperitoneally) for 14 days. Primary tumours were surgically removed at day 21. IRAK-inh treatment started 7 days before the primary tumour removal. (**d**) Representative images of IHC analysis of p-IRAK1 (S376) in mammary fat xenogfaft tumour tissues of control and IRAK-inh-treated mice. (**e**) Representative BLI images of NOD/SCID mice and BLI curves showing the lung metastases from day 0 to day 29, derived from lateral tail vein injection of indicated MDA231-LM2 cells. (**f**) Whole lung staining showing the metastatic nodules (upper) and *ex-vivo* BLI of lung metastasis (below) of representative mice at 6 weeks post injection. (**g**) IHC staining of indicated proteins in lung tissues in **f**. (**h**) Kaplan–Meier survival curves of mice from **f**. *n*=8. *P*-values were calculated with two-tailed *t*-test (**a**,**c**,**e**) or log-rank (Mantel–Cox) test (**h**).

To investigate whether IRAK1 is required for metastatic progression *in vivo*, we first assessed the effects of IRAK1 knockdown on TNBC xenograft mammary fat tumour growth and subsequent lung metastasis progression in NOD/SCID mice. To this end, we made use of the MDA231-LM2 cells expressing the *IRAK1* shRNA that targets the 3′-UTR of *IRAK1*, which allows functional rescue by ectopic *IRAK1*. The results showed that the primary tumour growth was markedly reduced in mice bearing tumours expressing *IRAK1* shRNA, compared with the vector control (Fig. 5a), whereby this knockdown effect was completely rescued in mice bearing tumours expressing both *IRAK1* shRNA and ectopic IRAK1 (Fig. 5a). IHC analysis of the harvested tumours indicated the corresponding changes of IRAK1 and IL-6 expressions in *IRAK1*-depleted tumours and in ectopic IRAK1-rescued tumours compared with control tumours, although cell proliferation marker Ki67 remained unchanged (Fig. 5b).

To assess lung metastasis, mammary fat tumours were removed after 21 days and the lung metastasis was assessed by lung bioluminescence imaging analysis. The effect of IRAK-inh on metastasis was also evaluated by administering the drug 7 days before the primary tumour removal and subsequent treatment for 14 days. As shown in Fig. 5c, *IRAK1* knockdown markedly reduced the lung metastasis and again the knockdown effect on metastasis was rescued by ectopic IRAK1. IRAK1-inh treatment also reduced lung metastasis burden (Fig. 5c) and markedly reduced the p-IRAK1 level in mammary pad xenograft tumour tissues (Fig. 5d). These findings indicate that IRAK1 is required for TNBC progression and that pharmacologic inhibition of IRAK1 is able to abolish metastatic progression.

To assess the effect of ectopic IRAK1 overexpression on lung metastasis, intravenous injection of MDA231-LM2 cells expressing vector control or ectopic *IRAK1* in NOD/SCID mice was performed. The result showed that ectopic IRAK1 drove a rapid formation of lung colonization and metastatic nodules as compared with the vector control, as determined by both *in vivo* and *ex vivo* lung bioluminescence imaging and whole lung staining (Fig. 5e,f). IHC analysis of the affected lungs indicated increased expression of IL-6 in tumours expressing ectopic *IRAK1* (Fig. 5g). As a result, mice bearing ectopic *IRAK1* suffered accelerated death compared with the control mice (Fig. 5h). Taken together, our results demonstrated through both loss- and gain-of-function studies a crucial role of IRAK1 in driving TNBC growth and metastasis.

### IRAK1 activation confers acquired resistance to paclitaxel

**Figure 6:**
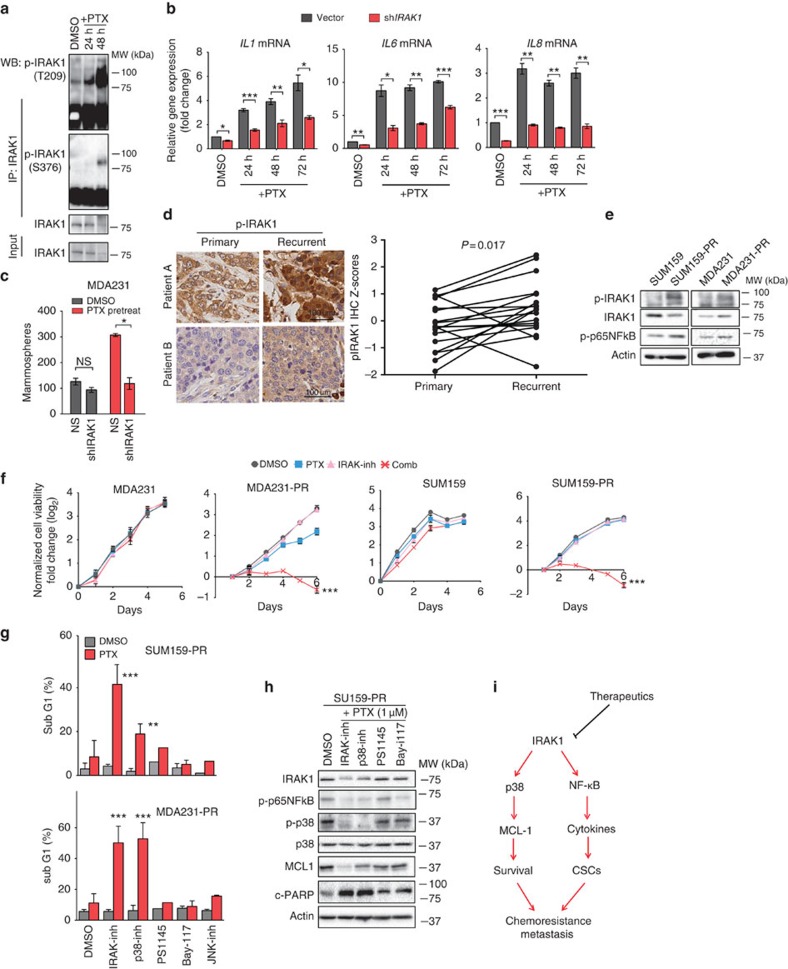
Role of IRAK1 signalling in acquired resistance to paclitaxel. (**a**) Western blotting showing the p-IRAK1 of immnoprecipitated IRAK1 in SUM159 cells treated with 5 nM paclitaxel (PTX) for 24 and 48 h. (**b**) Qantitative reverse transcriptase–PCR analysis of indicated cytokine mRNAs in MDA231 cells treated with 5 nM PTX for 24–72 h. (**c**) Mammosphere formation assay of MDA231 cells expressing vector or sh*IRAK1* after PTX (10 nM) pretreatment for 96 h in monolayer. PTX-treated cells were then washed and the viable cells were seeded for mammosphere assay. (**d**) IHC analysis of p-IRAK1 (S376) in primary and recurrent breast tumour samples. Left, representative IHC images. Scale bar, 100 μm. Right, quantification of p-IRAK1 levels in 18 paired tissue samples (paired two-tailed *t*-test). (**e**) Western blotting shows the indicated proteins in MDA231 and SUM159 parental and paclitaxel resistant (PR) sublines. (**f**) Cell viability of parental and paclitaxel resistant sublines were treated with respective doses of PTX, IRAK-inh (5 μM) or both. PTX, 0.43 and 75 nM for MDA2131 and MDA231-PR, respectively; 0.73 nM and 1 μM for SUM159 and SUM159-PR, respectively. (**g**) Apoptosis as determined by FACS analysis of Sub-G1 cells in SUM159-PR (top) and MDA231-PR (bottom) cells treated with paclitaxel, together with or without indicated small molecule inhibitors of IRAK1, p38, JNK or IKKβ/NF-κB (PS1145 and Bay117082). (**h**) Western blotting showing the indicated molecular signalling events in SUM159-PR cells treated in **g**. (**i**) Schematic representation of the roles of IRAK1 in driving metastasis and chemoresistance in TNBC. Error bars represent s.e.m, *n*=3. *P<0.05, **P<0.01, ***P<0,001. *P*-values were calculated with two-tailed *t*-test.

CSCs are regarded as crucial for relapse after chemotherapy^29^. It has also become evident that chemotherapy is able to induce CSCs repopulation through induction of inflammatory cytokines^30^,^31^,^32^. We next investigated whether IRAK1 signalling participates in paclitaxel-induced inflammatory induction and CSC enrichment. SUM159 treated with paclitaxel showed strong induction of IRAK1 phosphorylation at both T209 and S376 (Fig. 6a). Consistently, paclitaxel treatment resulted in strong induction of *IL1B*, *IL6* and *IL8* expression, which was markedly impaired on *IRAK1* knockdown (Fig. 6b). Interestingly, in contrast to paclitaxel, the other two chemotherapeutic agents adriamycin and cisplatin only induced modest or little responses in the cytokine induction (Supplementary Fig. 6A), indicating a prevalent role of IRAK1 in paclitaxel response.

To study a role of IRAK1 in chemotherapy-induced CSC repopulation, we treated MDA231 and SUM159 cells with 10 nM paclitaxel for 4 days, which resulted in a large number of floating dead cells. The dead cells were then washed off and the remaining viable cells were harvested for Aldefluor assay to measure aldehyde dehydrogenase (ALDH) activity, which has been previously shown to be relevant to CSCs of SUM159 and MDA231 (ref. 32). Alternatively, the viable cells after the paclitaxel treatment were seeded for mammosphere formation as a functional CSC assay. The result shows that paclitaxel treatment resulted in significant enrichments of ALDH^+^ cells (Supplementary Fig. 6B) and mammosphere formation (Fig. 6c), and this effect was significantly compromised on *IRAK1* knockdown (Fig. 6c) or IRAK-inh co-treatment (Supplementary Fig. 6C). These results indicate that IRAK1 signalling participates in chemotherapy response and contributes to chemotherapy-induced CSC enrichment.

Consistent with this *in vitro* observation, IHC analysis of 18 paired primary and chemo-recurrent tumour samples showed significantly increased p-IRAK1 expression in recurrent metastatic tumours compared with matched primary tumours, indicating a clinical association of p-IRAK1 with tumour recurrence (Fig. 6d).

Having shown a role of IRAK1 in paclitaxel response in TNBC, we next sought to determine whether IRAK1 activation contributes to TNBC acquiring resistance to paclitaxel. To do, we used MDA231 and SUM159 cell lines (express lower levels of IRAK1) and generated paclitaxel-resistant (PR) lines through stepwise exposure to increasing concentrations of paclitaxel over 3 months (Supplementary Fig. 7A). As anticipated, both of the resulting PR cell lines displayed increased p-IRAK1 and p-p65 NF-κB (Fig. 6e), indicating gain of activity of IRAK1–NF-κB signalling in TNBC cells upon acquisition of resistance to paclitaxel. Intriguingly, IRAK-inh, when combined with respective EC20 concentrations of paclitaxel, induced dramatic loss of cell viability in both PR cell lines but not in the respective parental cells (Fig. 6f), which was accompanied by massive apoptosis (Supplementary Fig. 7B). Interestingly, PR cells also showed significant cross-resistance to vincristine, another microtubule-targeting drug^33^, but not much to adriamycin and cisplatin (Supplementary Fig. 7C). Accordingly, IRAK-inh also sensitized vincristine for apoptosis induction in PR cells (Supplementary Fig. 7D). These results demonstrate a strong association of IRAK1 with microtubule-targeting agents and showed that therapeutic targeting of IRAK1 is able to circumvent paclitaxel resistance by inducing massive apoptosis.

We next investigated the molecular basis by which IRAK-inh sensitizes TNBC cells to paclitaxel-induced apoptosis. Although a combination of IRAK-inh and paclitaxel led to reduced secretion of IL-6, IL-8 and CXCL1 in PR cells (Supplementary Fig. 7E), treatments with two IκB kinase-β (IKKβ)/NF-κB inhibitors PS1145 and Bay117082 failed to sensitize paclitaxel to apoptosis (Fig. 6g). This suggests that in addition to NF-κB pathway, there are additional mechanisms of paclitaxel resistance linked to IRAK1-engaged modulation of apoptosis induction.

p38/JNK MAPKs are other downstream effectors of IRAK1 signalling that have been implicated in apoptosis modulation^34^,^35^,^36^. Unlike IKKβ/NF-κB inhibitors, we found that the p38 inhibitor was able to phenocopy IRAK-inh to sensitize paclitaxel-induced apoptosis in PR cells, although this effect was not evident for the JNK inhibitor (Fig. 6g). In line with these findings, both the IRAK-inh and p38 inhibitor, but not the two IKKβ/NF-κB inhibitors (PS1145 and Bay117082), were able to cooperate with paclitaxel to decrease p-p38 and MCl-1 expression, which was accompanied by increased poly (ADP-ribose) polymerase cleavage indicative of apoptosis (Fig. 6h). MCL-1 is known to be phosphorylated and stabilized by p38/JNK, to promote survival^37^. In addition, MCL-1 has been recently shown to be crucial for the viability of TNBC cells^38^. Thus, these findings along with the previous reports suggest that one possible mechanism by which IRAK-inh sensitizes paclitaxel for apoptosis induction is by acting to inhibit p38, which in turn decreases MCL-1 expresssion. However, we do not completely exclude the involvement of NF-κB pathway in this scenario.

Together, these findings suggest that both NF-κB and p38-MCL-1 signalling pathways downstream of IRAK1 can be attributed to the progressive development of paclitaxel. Although the former is perhaps mainly involved in repopulation of cells associated with CSC, the latter is relevant to promoting a survival mechanism to evade paclitaxel-elicited apoptosis. In light of these observations, we envision that therapeutic targeting of IRAK1 may therefore be a more effective strategy to eliminate both, as compared with inhibiting NF-κB alone, advocating a prime target for advanced metastatic TNBC (Fig. 6i).

Interestingly, traditional oriental medicine ginseng products ginsenoside Rb1 and its metabolite compound K (CK) have recently been reported to have the ability to inhibit IRAK1 and thus reduce inflammatory response^39^. We thus evaluated the two Ginsenosides for their capacity to inhibit TNBC aggressive growth and paclitaxel resistance. The results show that the CK compound, but not Rb1, was able to mimic the IRAK-inh to inhibit p-IRAK1 in MDA231-LM2 cells (Supplementary Fig. 8A), abolish MDA231-LM2 mammosphere growth (Supplementary Fig. 8B) and sensitize paclitaxel treatment for apoptosis (Supplementary Fig. 8C,D). Thus, for a translational point of view, this indicated a potential application of ginseng products to treat metastasis and chemoresistance in advanced TNBC patients with high levels of IRAK1, which might be worthy of further clinical exploration.

## Discussion

IRAK1 is an active kinase of the IL-1/TLR signalling pathway mainly involved in inflammation response. Although IL-1-signalling and TLR-MyD88 have been implicated in human cancers, IRAK1 alteration itself has not been previously linked to human malignancy until very recently^18^,^20^,^21^. We found that IRAK1 is overexpressed in a subset of breast cancers, mainly in TNBC, and pharmacologic inhibition of IRAK1 abolishes aggressive growth of TNBC and metastatic progression.

The ability of IRAK1 to affect TNBC progression appears to involve the regulation of NF-κB signalling as evidenced by reduced NF-κB reporter activity and NF-κB target expression on IRAK1 inhibition. It is known that NF-κB activation in CSC maintains stemness^40^,^41^. Moreover, NF-κB-related cytokines, such as IL-6, IL-8 and CXCL1, have a major role in TNBC tumorigenesis, including growth, metastasis, chemoresistance and CSC phenotypes^9^,^10^,^24^. We detected significant increased IRAK1 expression in metastatic clinical tumour samples compared with matched primary tumours. Intriguingly, this was recapitulated *in vitro* in MDA231, which expresses a low level of IRAK1, but showed increased expression and activity of IRAK1 when they become metastatic, and thus became much more sensitive to IRAK1 inhibition. These findings suggested that metastatic TNBC have a gain of IRAK1 dependency, thus highlighting the potential utility of therapeutic targeting of IRAK1 for metastatic disease. Indeed, we show that IRAK-inh effectively blocked the metatastic progression *in vivo* and extended the survival of the mice carrying TNBC metastasis. Although this study focused on the role of IRAK1-driven cytokine network in a cancer cell autonomous manner, IL-1β-mediated IRAK1 activation via tumour-infiltrating immune cell–tumour cell interaction may further enhance oncogenic activity of IRAK1 *in vivo*. We therefore expect that therapeutic targeting of IRAK1 may be particularly effective *in vivo* through abrogating both cancer-intrinsic and cancer-promoting immune response, although addressing this aspect will be technically challenging in immune-deficient mice.

Direct targeting of NF-κB signalling using IKKβ/RelA inhibitors has proven to be a challenge due to severe toxicity in patients^16^; thus, inhibiting IRAK1 as an alternative approach to target NF-κB in TNBC bears considerable implications for therapeutic treatment. Importantly, *IRAK1* knockout mice shows normal phenotype, which is in contrast to mice lacking RelA or other subunits of IKK complex that are embryonically lethal due to hepatic apoptosis^16^,^42^. Given the important role of NF-κB and its dependency on IRAK1 in TNBC progression, we reasoned that therapeutic targeting of IRAK1 might be able to achieve cancer selectivity, thus being a more accessible and less deleterious target than NF-κB itself for curtailing TNBC or other high IRAK1- and NF-κB-driven cancers.

Another major finding of this study is the identification of IRAK1 activation as a key driver event in acquired resistance to chemotherapy. Increasing evidence has begun to elucidate the crucial roles of chemotherapy-induced inflammatory cytokine or chemokine expression in CSC repopulation and possible tumour recurrence^10^,^20^,^30^,^31^,^43^. In TNBC, paclitaxel has been previously shown to induce cytokine secretion such as IL-6 and IL-8, through activation of various pathways including signal transducer and activator of transcription 3, transforming growth factor-β and hypoxia-inducible factor-1 (refs 32, 43, 44). Here we show that IRAK1 signalling is also activated by paclitaxel, which contributes to paclitaxel-induced cytokine expression and CSC enrichment. In line with these findings, we show that TNBC cells with acquired resistance to paclitaxel exhibited enhanced activity of IRAK1 and inhibition of IRAK1 is able to combat paclitaxel resistance by inducing massive apoptosis.

Of important note, the efficacy of IRAK1 inhibitor to enhance paclitaxel-induced apoptosis in our model is related to its ability to inhibit p38 MAPK activity, instead of NF-κB. Indeed, a p38 inhibitor similarly enabled paclitaxel to induce apoptosis in PR cells and both IRAK1 inhibitor and p38 inhibitor induced the downregulation of anti-apoptotic protein MCL-1. By contrast, inhibitors of IKKβ/NF-κB were insufficient to induce similar apoptotic response when combined with paclitaxel. This finding seems to be consistent with a recent report showing a crucial role for p38 in supporting the metastatic growth of TNBC cells^45^. In addition, depending on cellular context, MCL-1 can be phosphorylated and stabilized by p38 to promote anti-apoptotic effect^37^ and has been recently identified as a key survival factor to support the viability of TNBC cells^38^. Thus, we postulate that the gain of IRAK1-p38-MCL-1 dependency on acquired resistance to paclitaxel may constitute a key mechanism to withstand chemotherapy-induced apoptosis. Clearly, both NF-κB-related cytokine induction and p38 signalling are involved in paclitaxel resistance. However, there are major functional differences in their contributions to the acquired resistance process. Although the former is believed to be mainly involved in CSC expansion, the latter is more required to maintain the survival capacity.

From a translational point of view, therapeutic targeting of IRAK1 might be an effective therapeutic option for advanced metastatic TNBC, as it is sufficient to block both NF-κB and p38 signalling. Given that cytotoxic chemotherapy remains the standard of care for TNBC, our findings provide the rationale for developing more potent and drug-like small molecule inhibitors of IRAK1 kinase for targeting metastatic and recurrent TNBC tumours, to improve the efficacy of chemotherapy. In light of this view, our finding that the natural product ginsenoside compound (CK) is able to phenocopy the IRAK-inh to inhibit TNBC metastatic growth and combat chemoresistance is intriguing, as it can be readily tested in clinical trials for metastatic and chemo-refractory TNBC that express high levels of IRAK1.

## Methods

### Breast cancer molecular subtype and survival analysis

Cancer subtype-specific *IRAK1* gene expression analyses based on the gene expression of IRAK1–4 were performed on data generated by the The Cancer Genome Altas Research Network: http://cancergenome.nih.gov/. Kaplan–Meier survival analyses for disease outcomes were performed using the online database (www.kmplot.com) and the percentiles of the patients between the upper and lower quartiles were auto-selected based on the computed best performing thresholds as cutoffs. The *IRAK1* gene expression analysis on VandeVijver Breast Cohort^46^ and Schmidt Breast Cohort (GSE11121)^47^ analysis was performed on Oncomine data sets after patient stratification based on the metastasis events according to the author's original documentations.

### Cell cultures and viral infections

All cell lines were obtained from ATCC (Manassas, VA) within the last 5 years, except for MDA231-LM2 (a kind gift from Dr Yibin Kang, Princeton University)^27^. Cell lines authentication was performed by the ATCC using short tandem repeat DNA profiles and were tested negative for mycoplasma contamination. MDA231, BT549, MCF7, T47D, BT474, MB361, MB415, MB436, HS578T and MB157 breast cancer cell lines were grown in DMEM medium supplemented with 10% fetal bovine serum (FBS). SKBR3 cells were maintained in McCoy's 5A medium. HCC1806 and HCC1937 were maintained in RPMI medium supplemented with 10% FBS. HMEC and MCF10A normal breast epithelial cell line were purchased from ATCC and were grown in DMEM/F12 supplemented with 5% horse serum, 20 ng ml^−1^ epidermal growth factor (EGF), 0.5 mg ml^−1^ hydrocortisone, 100 ng ml^−1^ cholera toxin, 10 μg ml^−1^ insulin and penicillin/streptomycin (Invitrogen). All media were supplemented with 5,000 U ml^−1^ penicillin/streptomycin (Invitrogen). All cells were maintained at 37 °C with 5% CO_2_.

To generate IRAK1 shRNA cells, two different sequences (V3LHS_635467 and V3LHS_635469) from the GIPZ lentiviral shRNA system (Thermo Scientific, MA) were used to knock down IRAK1 constitutively in various TNBC cell lines. For inducible knockdown of IRAK1, an shRNA (V2THS-132369) from the TRIPZ Dox-inducible lentiviral shRNA system that targets the 3′-UTR region of IRAK1 was used as described (a gift from Dr Daniel T. Starczynowski, University of Cincinnati)^18^. Both constitutive and inducible shIRAK1 stable cells were maintained in complete medium supplemented with puromycin (1 μg ml^−1^), to select for positive clones. Dox (Clontech, Mountain View, CA) was used at a final concentration of 0.5 μg ml^−1^ for 48–72 h, to induce knockdown of IRAK1. The specific targeting sequences of shRNAs used to knock down IRAK1 are as summarized in Supplementary Table 1.

To generate the IRAK1 overexpressing plasmid (IRAK1 OE), complementary DNAs of IRAK1 (transcript 1, accession no: NM_001569.3) were amplified from normal breast tissue controls by PCR and inserted into the GFP-based expression vector pBabeMNires (PMN vector, a gift from LZ Penn, University of Toronto, Canada). QuikChange Multi Site-Directed Mutagenesis Kit (Agilent) was used to alter the lysine 239 residue to serine (K239S), rendering the kinase activity of IRAK1 inactive. To do so, primers were computed by the manufacturer's recommended tool QuikChange Primer Design^48^.

To establish paclitaxel-resistant TNBC cell lines, we treated SUM159 and MDA231 cells with increasing concentrations of paclitaxel over a period of 3 months. Due to the difference in the intrinsic EC_50_ to paclitaxel, 3.88 nM for SUM159 and 0.98 nM for MDA231, we started paclitaxel treatment at 5 nM and 0.5 nM, respectively. Once the cells had recovered from the treatment and exhibited normal growth rate, the paclitaxel concentration was increased by twofold. During this treatment cycle, only cells with high intrinsic resistance and those that had acquired resistance were able to survive. Treatment cycles continued until two stable paclitaxel-resistant cell lines were generated, SUM159-PR and MDA231-PR, which were stably maintained in 1 μM and 75 nM paclitaxel in complete medium, respectively.

### Reagents and antibodies

Recombinant IL-1β, IL-6, IL-8 and CXCL1 were purchased from Peprotech (Rocky Hill, NJ). Paclitaxel (catalogue number: T7402), Vincristine (catalogue number: V8879), Doxorubicin (catalogue number: D1515), Cisplatin (catalogue number: P4394) and IRAK1/4 inhibitor (catalogue number: I5409) were purchased from Sigma-Aldrich (St Louis, MO). SCIO 469 (p38 inhibitor, catalogue number: 1671), PS1145 (IKK inhibitor, catalogue number: 1568), BAY-11–7082 (IKK inhibitor and anti-inflammatory, Cat no: 2132) and AEG 3482 (JNK inhibitor, catalogue number: 1291) were purchased from Axon Medchem (Reston, VA). CK, a metabolite of Ginsenoside Rb1, was purchased from Chengdu Must Bio-Technology Co. (Chengdu, China).

The following antibodies were used for western blot analysis: anti-IRAK1 (catalogue number: #4359, 1:500 dilution), anti-cleaved poly (ADP-ribose) polymerase (catalogue number: #9541, 1:200 dilution), anti-p38 MAPK (catalogue number: #9212, 1:1000 dilution), anti-phospho-p38 MAPK (T180/Y182) (catalogue number: #9211, 1:1000 dilution) and anti-phospho-NF-κB p65 (S536) (catalogue number: #3031, 1:1000 dilution) were purchased from Cell Signaling (Danvers, MA). Anti-phospho-IRAK1 (T209) was purchased from Assay Biotech (Sunnyvale, CA; catalogue number: A1074; 1:1000 dilution) and anti-phospho-IRAK1 (S376) from Genetex (Irvine, CA; catalogue number: GTX60194; 1:500 dilution). Anti-MCL-1 was purchased from Santa Cruz Biotechnology (Santa Cruz, CA; catalogue number: sc-819, 1:1000 dilution). Anti-actin was purchased from Sigma-Aldrich (catalogue number: A5441, 1:20000 dilution). Detection of bands was performed with the ChemiDoc MP Imaging Systems (Bio-Rad, Hercules, CA) and bands were subjected to further densitometric analysis with Image Lab software (Version 4.1, Bio-rad).

### Quantitative PCR analysis

Total RNA was isolated by using Qiazol (Life Technologies, Carlsbad, CA) and purified with the RNeasy Mini Kit (Qiagen, Valencia, CA). Reverse-transcription and quantitative PCR assays were performed using High Capacity cDNA Archive kit and KAPA SyBr Fast qPCR kit (KAPA Biosystems, Wilmington, MA). For quantification of mRNA levels, 18S level was used as an internal control. All reactions were analysed in an Applied Biosystems PRISM 7500 Fast Real-Time PCR system in 96-well plate format. Real-time primer sequences shown in Supplementary Table 2.

### Co-immunoprecipitation

The whole-cell lysates were extracted with the NE-PER kit (Pierce Biotechnology) and subjected to immunoprecipitation using 2 μg total-IRAK1 antibody (Santa Cruz Biotechnology; catalogue number: sc-7883). The precipitated protein complex was captured using protein A-Agarose beads (Roche, Indianapolis, IN, USA) and extensively washed with the washing buffer (50 mM Tris-HCl, 150 mM NaCl, 0.1% Triton). The precipitated proteins were dissolved in SDS sample buffer along with 3 mM dithiothreitol and subjected to immunoblotting analysis. Antibodies for phospho-IRAK1 (T209) and (S376) were used for detection of IRAK1 phosphorylation.

### ELISA assay and cytokine antibody array

IL-6, IL-8 and CXCL1 levels were assessed using ELISA assay kit (Boster Bio, Pleasanton, CA). Supernatants of the mammosphere formation assay were collected after 10 days in culture. Dilution (1:50) was performed on the supernatants before quantifying the amount of cytokines according to the manufacturer's protocol. For cytokine Ab array, supernatants collected from the non specific (NS) or shIRAK1 MB436 mammospheres after 10 days in culture were used directly without further dilution. Semi-quantitative detection of 120 human cytokines and chemokines in the supernatants were performed using the RayBio C-series Human Cytokine Antibody Array C1000 (RayBiotech, Inc., Norcross, GS; catalogue number: #AAH-CYT-1000-2) as per the manufacturer's instructions. Detection of dots was performed with the ChemiDoc MP Imaging Systems (Bio-Rad) and the intensity of dots were quantified by densitometric analysis using the ImageJ software^49^. The raw numerical densitometry data were extracted and subjected to background subtraction before normalizing the signal for each cytokines against the positive control signals in each cytokine array.

### Cell proliferation assay and flow cytometry

For cell proliferation assay, the optimal cell seeding was first determined empirically for all cell lines by examining the growth of a wide range of seeding densities in a 96-well format, to identify conditions that permitted proliferation for 7 days. Cells were then plated at the optimal seeding density 24 h before small interfering RNA or drug treatment in triplicate. Plates were incubated for 7 days at 37 °C in 5% CO_2_. Cells were then lysed with CellTiter-Glo (Promega, Madison, WI) and chemiluminescent signal was detected with a microplate reader on days 0, 1, 3, 5 and 7. In addition, an untreated plate of cells was harvested at the time of drug or small interfering RNA addition (*T*_0_), to quantify the starting number of cells. CellTiter-Glo values obtained after the 7-day treatment were expressed as percentages of the *T*_0_ value and plotted against time of treatment.

Cell cycle analysis was done by DNA content quantification to quantify the sub-G1 population, which is reflective of the extent of cell death. Briefly, the cells were fixed with 70% ethanol and stained with propidium iodide (50 μg ml^−1^) staining. The stained cells were analysed by FACScalibur (BD Biosciences, Singapore) and quantified by using CellQuest software (BD Biosciences).

### 3D Matrigel assay

Eight-well chamber slides (Falcon, Dallas, TX; catalogue number: 354656) were precoated with 7.6 mg ml^−1^ growth factor-reduced Matrigel (Falcon; catalogue number: 354230) for 30 min at 37 °C. Approximately 5 × 10^3^ for MDA231, 1 × 10^4^ for MB436 and MB468, and 1.5 × 10^4^ cells for BT549 with indicated treatments were seeded in each well with DMEM containing 10% (vol/vol) FBS and 150 μg ml^−1^ Matrigel. Medium were replenished every 3 days and cell growth was monitored every 3 days by imaging over duration of 10–14 days.

### Mammosphere formation assay

Active growing cells were treated with 0.05% trypsin for 10 min then passed through 0.4 μm cell strainer, to achieve single cell suspension. Cells were plated (MDA231: 1 × 10^4^; MB436: 3 × 10^4^; MB468: 4 × 10^4^ cells per well, BT549: 3 × 10^4^, MDA231-LM2: 3 × 10^4^) seeded in six-well ultra-low attachment plates (Corning, Corning, NY, cat: CLS3471) in Mammocult medium (Stem Cell Technologies, Vancouver, BC, Canada), supplemented with fresh hydrocortisone (0.5 μg ml^−1^) and heparin (1:500). Tumorspheres were cultured for 7 days before being counted and photographed. Imaging and quantification were done using GelCount apparatus and associated software (Oxford Optronix, Abingdon, UK). For serial passages of tumorsphere formation assay, the spheres were collected by gentle centrifugation, dissociated to single cells for passaging tumorspheres every 14 days and counted. Tumorspheres were photographed and quantified 7–12 days later using a GelCount Colony Counter (Oxford Optronix) after staining with INT (2-(4-iodophenyl)-3-(4-nitrophenyl)-5-phenyl-2H-tetrazolium chloride, Sigma-Aldrich).

For established PDX tumour-derived mammopheres, tumours were surgically removed and washed with PBS-supplemented Antibiotic–Antimycotic (Invitrogen; catalogue number: 15240-062). Next, the samples were minced with a sterile blade and digested in 1 mg ml^−1^ collagenase/dispase (Roche) in DMEM/F12 medium at 37 °C in an incubator for 3 h. After incubation, the cell suspensions were triturated by passing through 70 and 40 μm cell strainers (BD Falcon, San Jose, CA, USA). Cells were collected by centrifugation before being plated in six-well ultra-low attachment plate (Corning) in a serum-free medium containing DMEM/F12 (1:1) (Gibco), supplemented with B27 (Invitrogen; catalogue number: 12587-010), N2 (catalogue number: 17502-048), 20 ng ml^−1^ EGF and 20 ng ml^−1^ β-fibroblast growth factor (BD Biosciences), and 4 μg ml^−1^ heparin (Sigma-Aldrich) and Antibiotic–Antimycotic at density of 20,000 cells per well. After 12–15 days tumorspheres were passaged by accutase (Invitrogen, A1110501) digestion followed by replating in the same manner as previous generation. For drug treatment, drugs and equal volume solvent controls were added to the cells at the time of seeding.

### Anchorage-independent colony formation assay

Experiments were carried out in six-well plates coated with a base layer of DMEM containing 0.6% agar, cells were seeded at a density of 10,000 cells per well in DMEM containing 0.3% agar, 10% FBS for 14 days. Colonies were stained with iodonitrotetrazolium chloride (INT, Sigma, St Louis, MO) overnight. The number and size of colonies were analysed using GelCount (Oxford Optronix) according to the manufacturer's instruction.

### Transwell invasion assay

Transwell invasion assay were performed using 24-well FluoroBlok transwell insert (Falcon) with a pore size of 8 μm, according to the manufacturer's protocol. In brief, the inserts were pre-coated with growth factor-reduced Matrigel (BD Biosciences, catalogue number: 354230 Falcon) for 6 h at 37 °C at the concentration of 600 μg ml^−1^. Then, 5 × 10^4^ of cells were seeded into each insert in DMEM containing 0.25% FBS as the serum starvation medium. DMEM supplemented with 0.5% FBS and 100 ng ml^−1^ EGF was added outside the chamber as chemo-attractant. Invaded cells were fixed after 48 h of incubation by using 3.7% formaldehyde and stained with 25 μg ml^−1^ propidium iodide (Sigma-Alrich). Ten fields per insert were scanned and numbers of invaded cells were counted with Cellomics ArrayScan.

### ALDEFLUOR assay

ALLEFLUOR assay was performed using the manufacturer's recommended protocol (ALDEFLUOR kit, Stemcell Technologies; catalogue number: #01700). In brief, one million single-cell suspensions were centrifuged and resuspended in ALDEFLUOR assay buffer supplied in the kit. Each sample cells were incubated with or without an ALDH-specific inhibitor 15 μM diethylaminobenzaldehyde in the presence of 0.15 μM ALDH substrate. ALDEFLUOR stainings were detected using fluorescein isothiocyanate channel of a FACSCalibur Flow Cytometry System (BD Biosciences) after 25 min incubation at 37 °C. Diethylaminobenzaldehyde inhibitor control sample was used as sorting gate reflecting background fluorescence levels for each cell lines.

### Dual luciferase reporter assay

NF-κB-specific reporter plasmid pGL4.32 and its negative control pGL4.15 were purchased from Promega. Cells were harvested 48 h after transfection and analysed with the Dual Luciferase system (Promega) according to the manufacturer's protocol. To analyse luciferase activity, firefly signals of pGL4.32/pGL.15 were normalized to *Renilla* signals of pRL null in respective samples. pGL4.32/pRL-null ratio were further normalized to pGL4.15/pRL-null ratio, to obtain normalized values corrected for the changes of basic transcription activity for indicated treatment of the cells.

### Animal studies

Six to eight week old female NOD/SCID mice were purchased from InVivos (Singapore). Based on power calculation (http:/www.biomath.info/power/index.htm), six to ten mice per group were sufficient to detect significant difference with a high statistical power. For the subcutaneous xenograft model, 7.5 × 10^6^ MB436 cells and 5 × 10^6^ MDA231 carrying Dox-inducible shIRAK1 (*n*=12 for each cell line) were mixed with Matrigel (BD Biosciences; catalogue number: 354234) at 1:1 ratio in a 50-μl total volume. Dox (100 mg kg^−1^, BD) (Clontech) was administered via oral gavage, to induce IRAK1 knockdown in the shIRAK1 group (*n*=6), whereas the control group was given PBS (*n*=6). Tumours were measured by vernier caliper and the tumour volume was calculated with the following formula: *V*=*W* × *W* × *L*/2.

For the orthotropic mammary fat pad model, 1 × 10^6^ MDA231-LM2 cells carrying control vector, shIRAK1 and ectopic IRAK1 rescue (*n*=8 each group) were mixed with Matrigel (BD Biosciences; catalogue number: 354234) at 1:1 ratio in a 20-μl total volume. Cells were engrafted in mammary fat of 6- to 8-week-old female NOD/SCID mice on day 0. Mice bearing the xenograft of the control group were further randomized and treated with either vehicle or IRAK-inh (4 mg kg^−1^, *n*=6) daily from day 20 to day 37 via intraperitoneal injection. Primary mammary tumours were measured by vernier calliper and the tumour volume was calculated with the following formula: *V*=*W* × *W* × *L*/2 before surgically harvesting the primary tumours on day 27. All the tumour size measurements were done by two persons, with one person blinded to the treatment group. Tumour size showing more than twice the s.d. of the mean at the time of randomization were excluded for analysis.

Lung metastasis development was monitored weekly by bioluminescence imaging. For *ex-vivo* imaging of the whole lung, mice were first killed via carbon dioxide inhalation and the lungs were harvested immediately. The lungs were submerged individually in 150 μg ml^−1^ of Promega VivoGlo Luciferin in PBS for 5 min before imaging with IVIS Imaging System (Xenogen, Alameda, CA).

For the tail vein xenograft model, 1 × 10^5^ MDA231-LM2 cells carrying control vector and IRAK overexpression (*n*=10 each group) were injected via lateral tail vein in 6- to 8-week-old female NOD/SCID mice. Lung metastasis development was monitored weekly by bioluminescence imaging.

For bioluminescence imaging, 150 mg kg^−1^ of Promega VivoGlo Luciferin in PBS were given to mice intraperitoneally and imaged with IVIS imaging System (Xenogen) until 6 weeks post injection. The photon values were recorded using Living Image 3.1. Differences among groups and treatments were determined by analysis of variance followed by Student's *t*-test (***P<0.001; NS, not significant). Error bars represent means±s.e.m. Animal survival curve was generated using Kaplan–Meier analysis and the statistical parameters were calculated by log-rank (Mantel–Cox) test using Graphpad Prism software (Version 6.0).

### IHC staining for xenograft tumours

Harvested tissues were fixed in 10% formalin solution, (HT501128, Sigma-Alrich). Tissues were dehydrated and embedded in paraffin. Paraffin-embedded tissue sections (5 μm thick) were cut, deparaffinized and rehydrated, and antigens were retrieved using pH 6 sodium citrate. The sections were then incubated in 0.06% hydrogen peroxide at room temperature, to block endogenous peroxidase. The slides were incubated overnight with anti-IRAK1 (catalogue number: sc-7883, 1:100 dilution), anti-vimentin (catalogue number: sc-6260, 1:500 dilution) from Santa Cruz Biotechnology and anti-IL6 from Abcam (Cambridge, MA; catalogue number: ab1543670, 1:1000 dilution) overnight, followed by 60 min incubation with anti-mouse IgG/rabbit IgG (catalogue number: PK-6200, 1:2000 dilution) and 60 min of Avidin DH and Biotinylated Horseradish Peroxidase H. ImmPACT DAB Peroxidase Substrate (catalogue number: SK-4105) was used as the chromogen. Vector Hematoxylin QS (H-3404) was used as counterstain.

### IHC staining for TMA and clinical samples

Breast Cancer Tissue microarray slides BR1505 and IMH-364 were purchased from US Biomax (Rockville, MD) and Novus Biologicals (Littleton, CO), respectively. Paraffin-embedded sections of primary and recurrent tumour were obtained from Tan Tock Seng Hospital, Singapore, and John Wayne Cancer Institute (CA, USA). Staining and image analysis of TMA and the clinical samples were performed by Histopathology Department of the Institute of Molecular and Cell Biology, Agency for Science, Technology, and Research (A*STAR), Singapore. Briefly, paraffin-embedded tissue sections and the TMAs were deparaffinized, rehydrated and antigens were retrieved by proteinase K solution; sections were then incubated in 3% H_2_O_2_ at room temperature, to block endogenous peroxidase. Slides were incubated in total-IRAK1 Ab from Santa Cruz Biotechnology (catalogue number: sc-7883, 1:1000 dilution) or phospho-IRAK1 (S376) Ab from Genetex (catalogue number: GTX60149, 1:500 dilution) for 45 min, followed by 30 min incubation with anti-mouse Labelled Polymer (Dako, CA).

Specificity of the immunostaining was determined by the inclusion of isotype-specific IgG as a negative control. The detection system was DAB+ Substrate-Chromogen Solution (Dako). The sections were counterstained with haematoxylin. Slides were scanned at × 20 using a Leica SCN400 slide scanner (Leica Microsystems, Germany). Images were exported to Slidepath Digital Image Hub (Leica Microsystems) for viewing. TMA cores were analysed using the Measure Stained Cells algorithm of Slidepath Tissue IA software (Leica Microsystems). The total cellular H-score were then further normalized and expressed as *Z*-score after conversion with the following formula, *z*=(total cellular H-score of each tumour−mean H-score)/s.d. of all tumours. Data were collated using Microsoft Excel. Scanning and image analysis was performed by the Advanced Molecular Pathology Laboratory, IMCB, Singapore.

### Study approval

Human tissue samples were provided from Tan Tock Seng Hospital, John Wayne Cancer Institute and Odense University Hospital (Denmark). Studies with these samples were approved by their institutional review boards. Informed written consent had been previously obtained from each individual who agreed to provide tissue for research purposes. All animal studies were conducted in compliance with animal protocols approved by the A*STAR-Biopolis Institutional Animal Care and Use Committee of Singapore.

### Statistical analyses

All *in-vitro* experiments were repeated at least three times, unless stated otherwise, and data are reported as mean+s.e.m. To normalize the expression of each patient cohorts, expression values were normalized by calculating the *z*-score for each independent data set, the differences were assessed by two-tailed Student's using Student's *t*-test or one-way analysis of variance for multiple group comparisons using GraphPad Prism 6 software. Animal study survival curves were plotted using Kaplan–Meier analysis and the statistical parameters were calculated by log-rank (Mantel–Cox) test using Graphpad Prism. In all statistical tests, the resulting *P*≤0.05 was considered significant unless stated otherwise.

## Additional information

**How to cite this article:** Wee, Z. N. *et al*. IRAK1 is a therapeutic target that drives breast cancer metastasis and resistance to paclitaxel. *Nat. Commun*. 6:8746 doi: 10.1038/ncomms9746 (2015).

## Supplementary Material

Supplementary Information Supplementary Figures 1-9 and Supplementary Tables 1-2
