# Correction: Corrigendum: IRAK1 is a therapeutic target that drives breast cancer metastasis and resistance to paclitaxel

**DOI:** 10.1038/ncomms10054

**Published:** 2015-11-20

**Authors:** Zhen Ning Wee, Siti Maryam J. M. Yatim, Vera K. Kohlabauer, Min Feng, Jian Yuan Goh, Yi Bao, Puay Leng Lee, Songjing Zhang, Pan Pan Wang, Elgene Lim, Wai Leong Tam, Yu Cai, Henrik J. Ditzel, Dave S. B. Hoon, Ern Yu Tan, Qiang Yu

10.1038/ncomms9746

The original version of this Article contained an error in the spelling of the author Yi Bao, which was incorrectly given as Bao Yi. This has now been corrected in both the PDF and HTML versions of the Article.

