# Supplementary material for: IRAK1 is a therapeutic target that drives breast cancer metastasis and resistance to paclitaxel
Source: Nat Commun. 2015 Oct 27;6:8746. doi: 10.1038/ncomms9746 (PMC4640083; doi:10.1038/ncomms9746)
Supplement: Supplementary Information — Supplementary Figures 1-9 and Supplementary Tables 1-2 [file ncomms9746-s1.pdf]

Supplementary Figures 1-9 and Supplementary Tables 1-2

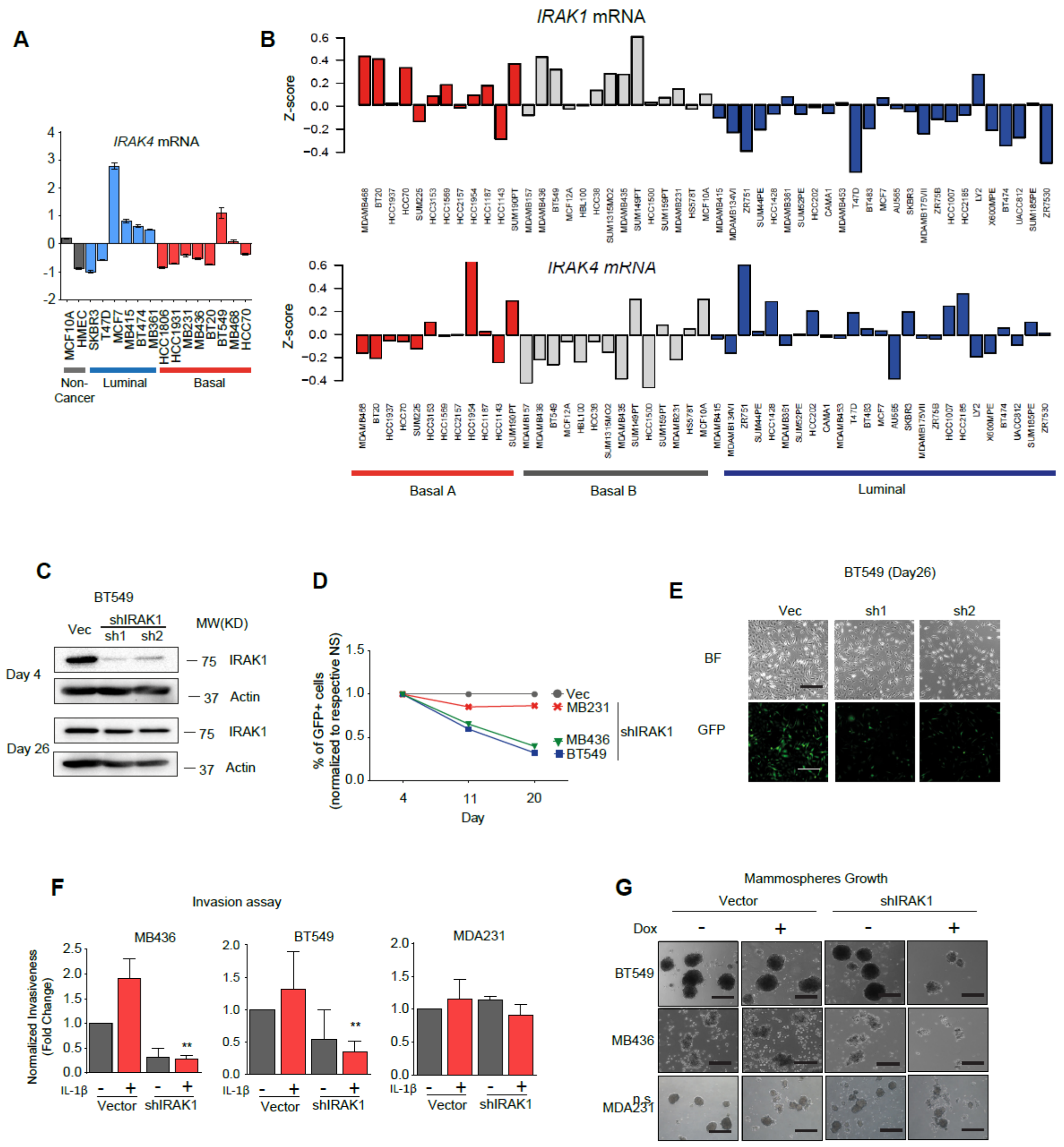

Supplementary Figure 1. IRAK1 knockdown impairs aggressive growth of TNBC cells

(A) RT-PCR analysis showing the expression of *IRAK4* mRNA in indicated breast cancer cell lines. **(B)** Expression profiles of *IRAK1* and *IRAK4* mRNAs in 51 breast cancer cell lines using the GOBO database. (**C**) Western blot showing IRAK1 expression in BT549 cells expressing non specific vector control (vector) or shIRAK1 sequence 1 and 2 (sh1 and sh2). **(D)** Percentage of GFP positive cells over time in TNBC expressing vector control (vector) or shIRAK1. **(E)** Microscope images of indicated BT549 cells at day 26. scale bars, 100 μm. (**F**) Invasion assay of TNBC cells in response to IL-1β treatment for 3 days. Error bars represent s.e.m, n=3. * *P*<0.05, student T -test. n.s, no significant. **(G)** Microscope images of mammosphere cells treated as indicated. scale bars, 100 μm

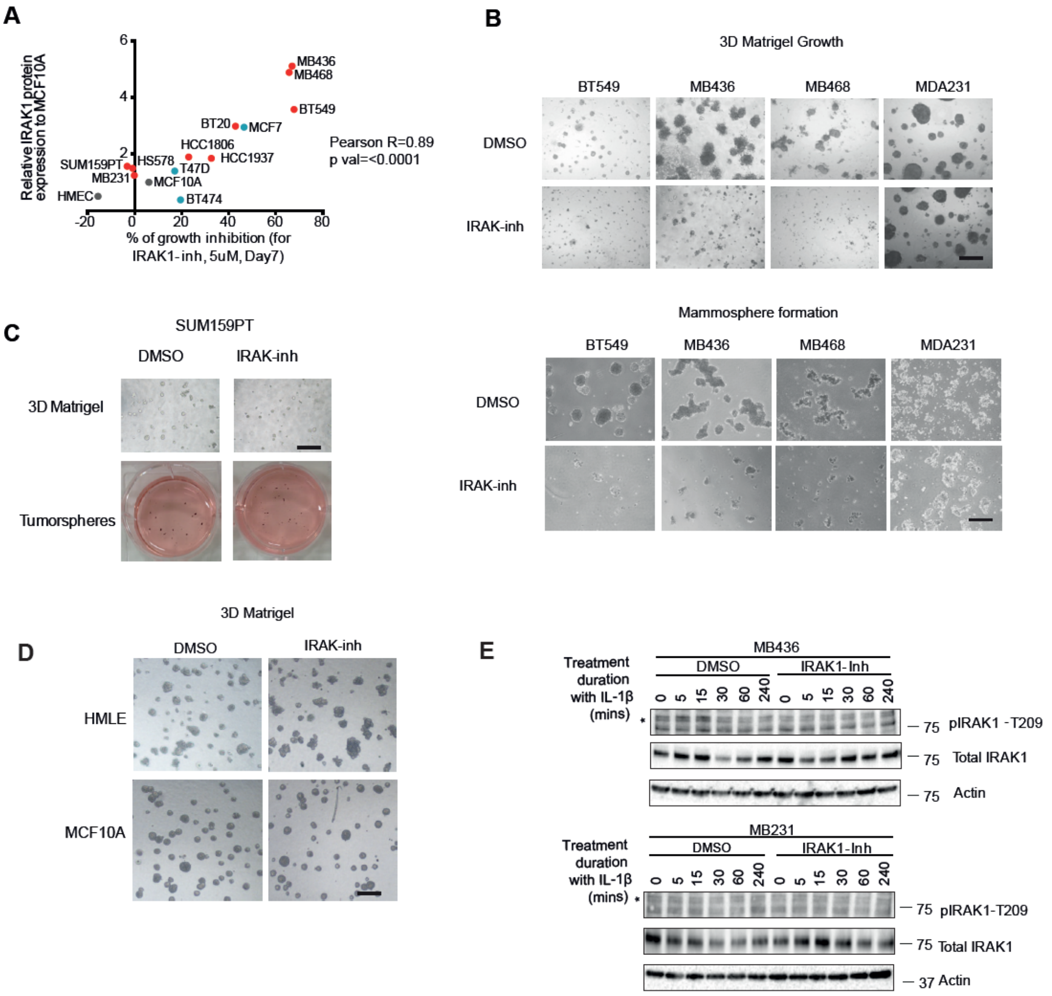

Supplementary Figure 2. Effects of IRAK1-inhibitor treatment on TNBC

(A) Spearman's correlation analysis of IRAK1 protein expression and their sensitivity to IRAK-inh treatment in the cell viability assay as represented by growth inhibition (%). IRAK1 protein expression was quantified by densitometry as shown in Figure 2B and normalized to the IRAK1 expression of MCF10A. **(B)** Representative microscope images of 3D Matrigel and mammosphere growth treated with 5 μM IRAK-inh for one week. Scale bars, 100 μm. (**C**) Representative microscope images of 3D Matrigel and mammosphere growth of SUM159PT cells treated with 5 μM IRAK-inh for one week. Scale bars, 100 μm. **(D)** Representative microscopy images of 3D Matrigel growth assay showing the morphology of non-cancerous MCF10A and HMLE cell lines after one week of IRAK-inh (5 μM) treatment. Scale bars, 100 μm. **(E)** Western blot analysis of total and p-IRAK1 at indicated time points after IL-1β (10 ng/ml) and IRAK-inh (5 μM) treatment on MB436 and MDA231 cell lines. Actin, loading control.

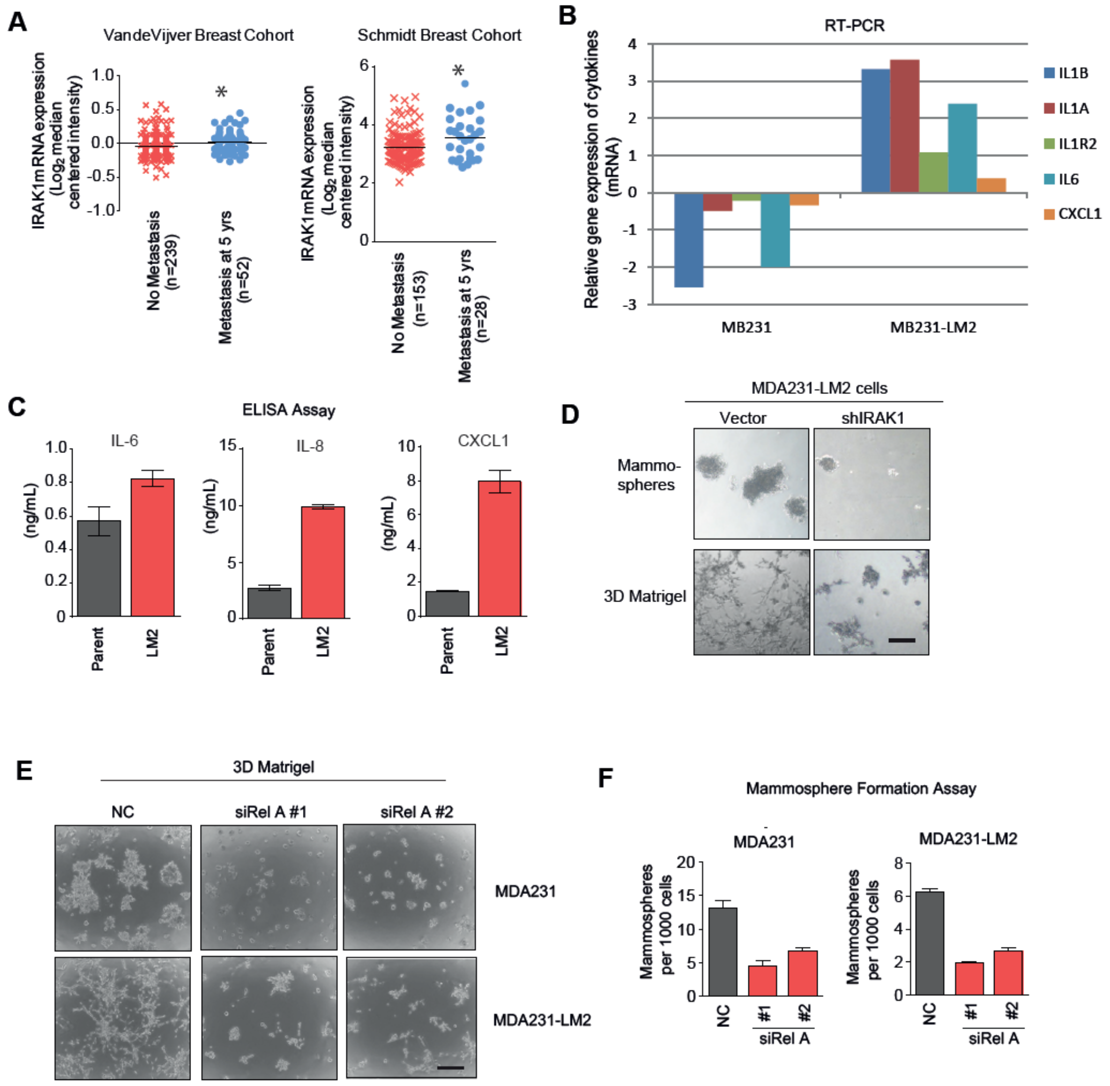

Supplementary Figure 3. TNBC metastatic cells shows increased IRAK1 signaling and IRAK1-dependency.

(A) Stratified analysis of Van de Vijver Breast Cohort and Schmidt Breast Cohort correlating *IRAK1* levels with 5-year metastasis incidence in breast cancer patients. **(B)** q-PCR analysis of indicated cytokines mRNAs in MDA231 and MDA231-LM2 cells. **(C)** ELISA assay of indicated cytokine levels in conditioned medium of MDA231 and MDA231-LM2 cells. **(D)** 3D Matrigel and mammosphere growth of MDA231-LM2 cells expressing vector or shIRAK1. Scale bars, 100 µm. **(E and F)** Quantifications of 3D Matrigel and mammosphere formation assay of MDA231 and MDA231-LM2 cells after RelA knock down. Scale bars, 100 µm. Error bars represent s.e,m, n=3. * *P*<0.05, student T-test.

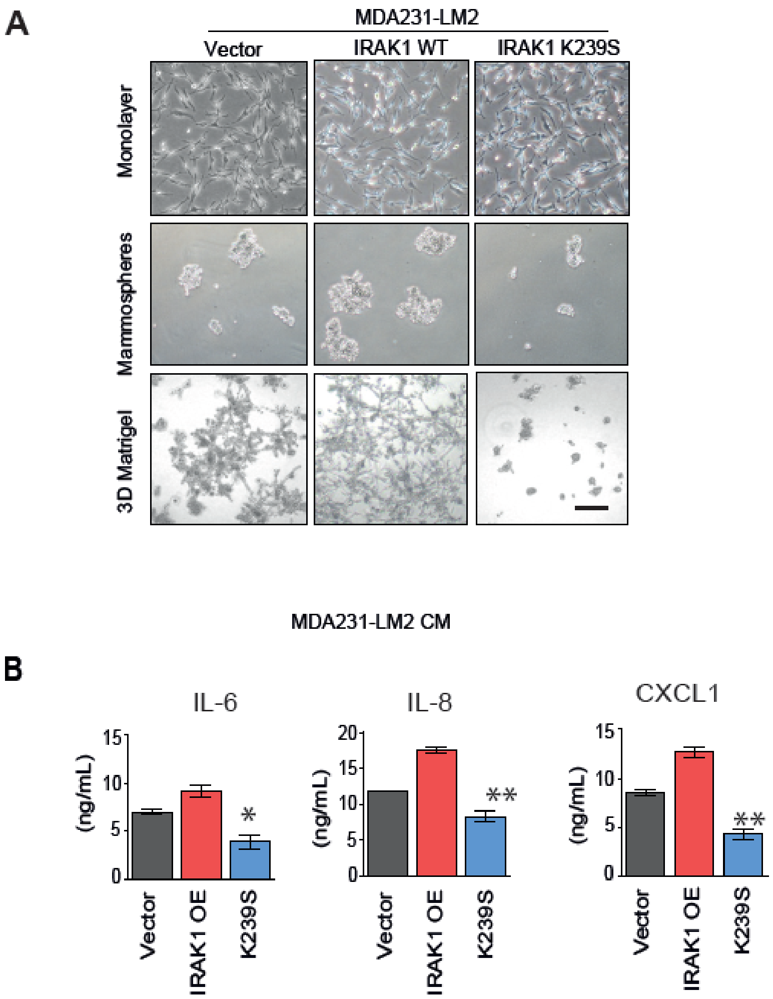

Supplementary Figure 4. IRAK1 kinase activity is required for the growth of metastatic MDA231-LM2 cells

(A) Monolayer, 3D Matrigel and mammosphere growth of MDA231-LM2 cells expressing vector, ectopic IRAK1 and kinase-dead mutant IRAK1. Scale bars, 100 μm. **(B)** ELISA assays of indicated cytokines in conditional medium of MDA231-LM2 cells in (**A**). Error bars represent s.e,m, n=3. * *P*<0.05, ***P*<0.01, student T-test.

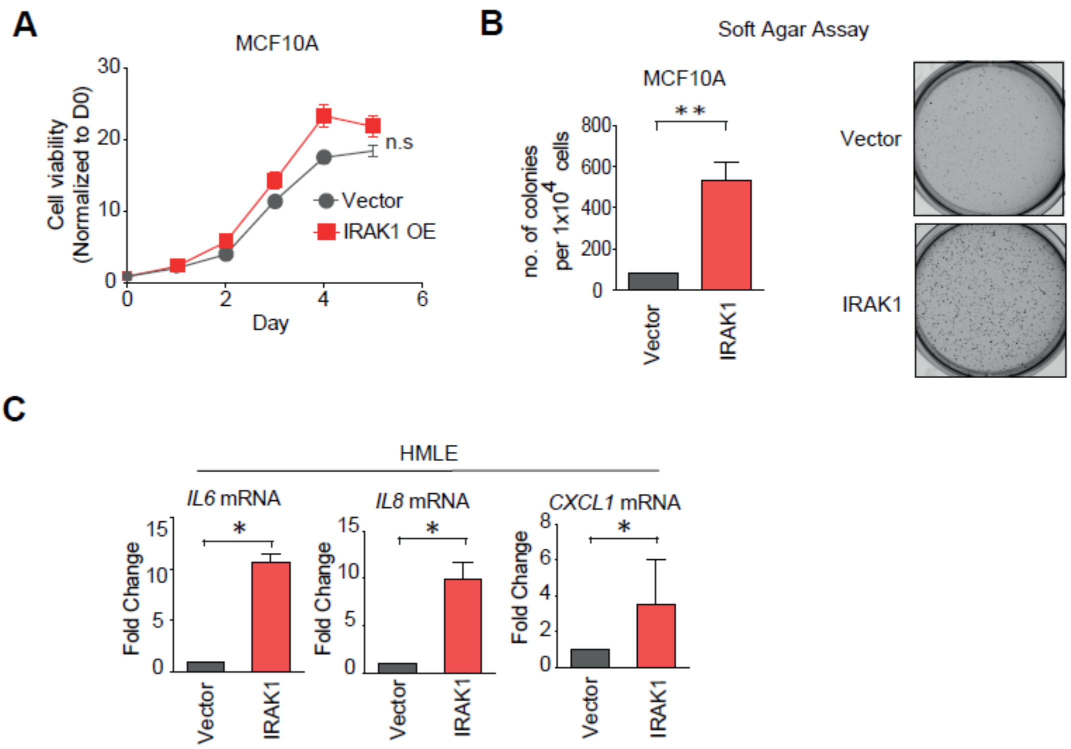

Supplementary Figure 5. Ecotopic IRAK1 is sufficient to drive aggressive growth of non-cancerous mammary epithelial cells.
(A) Cell proliferation of MCF10A cells overexpressing either the empty vector or ectopic IRAK1. **(B)** Soft agar assay. **C)** qRT-PCR analysis of *IL6*, *IL8* and *CXCL1* mRNAs in indicated cells treated with IL-1β (10 ng/ml). Error bars represent s.e.m, n=3. *P<0,05, **P<0.05, n.s, no significant, student T-test.

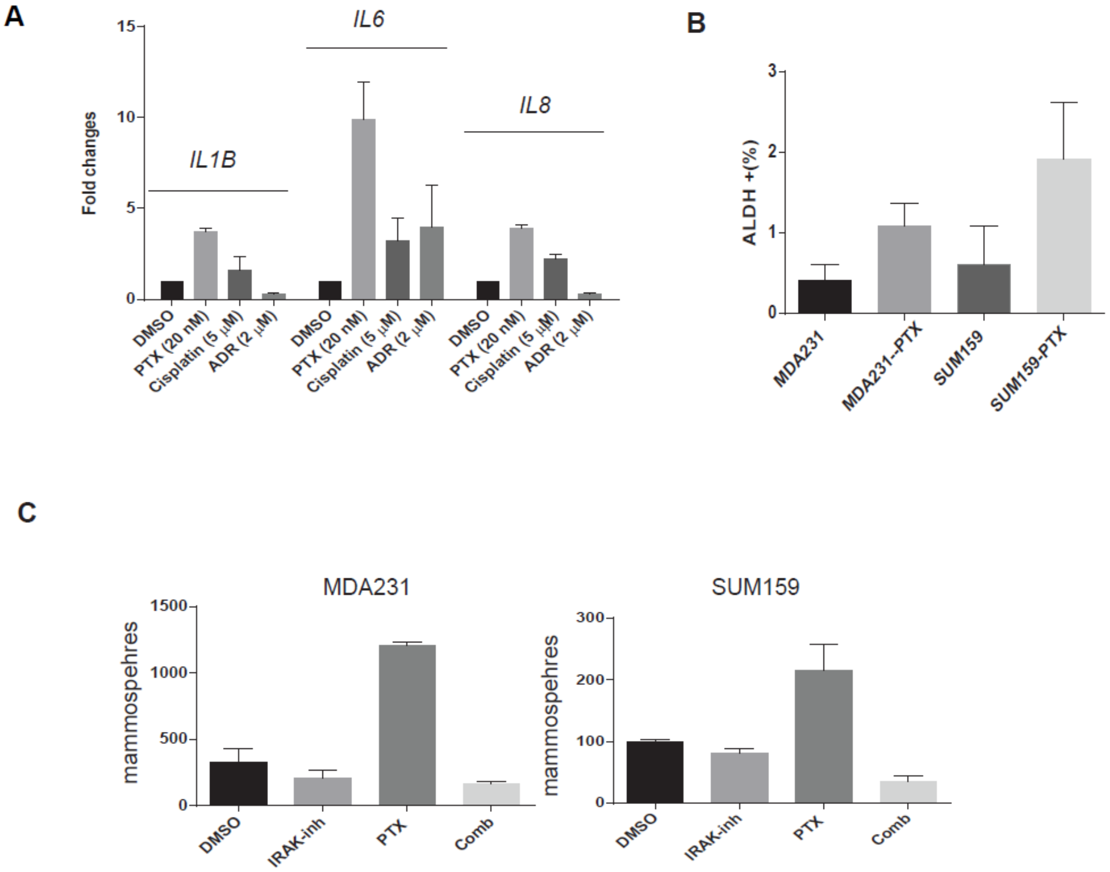

Supplementary Figure 6. IRAK1 activation contributes to paclitaxel-induced cytokine induction and CSC enrichment

(A) qRT-PCR analysis of cytokine expression in response to treatment with various chemotherapeutic agents in MDA231 cells. (B) ALDH assay of indicated cells treated with paclitaxel (10 nM) for 4 days. (**C**) Mammosphere formation of indicated cells treated with paclitaxel (10 nM) alone or co-treated with IRAK-inh (5 μM). For (B) and (C), floating dead cells were washed off and the remaining viable cells were harvested for ALDH assay or mammosphere formation assay. Error bars represent s.e.m, n=3.

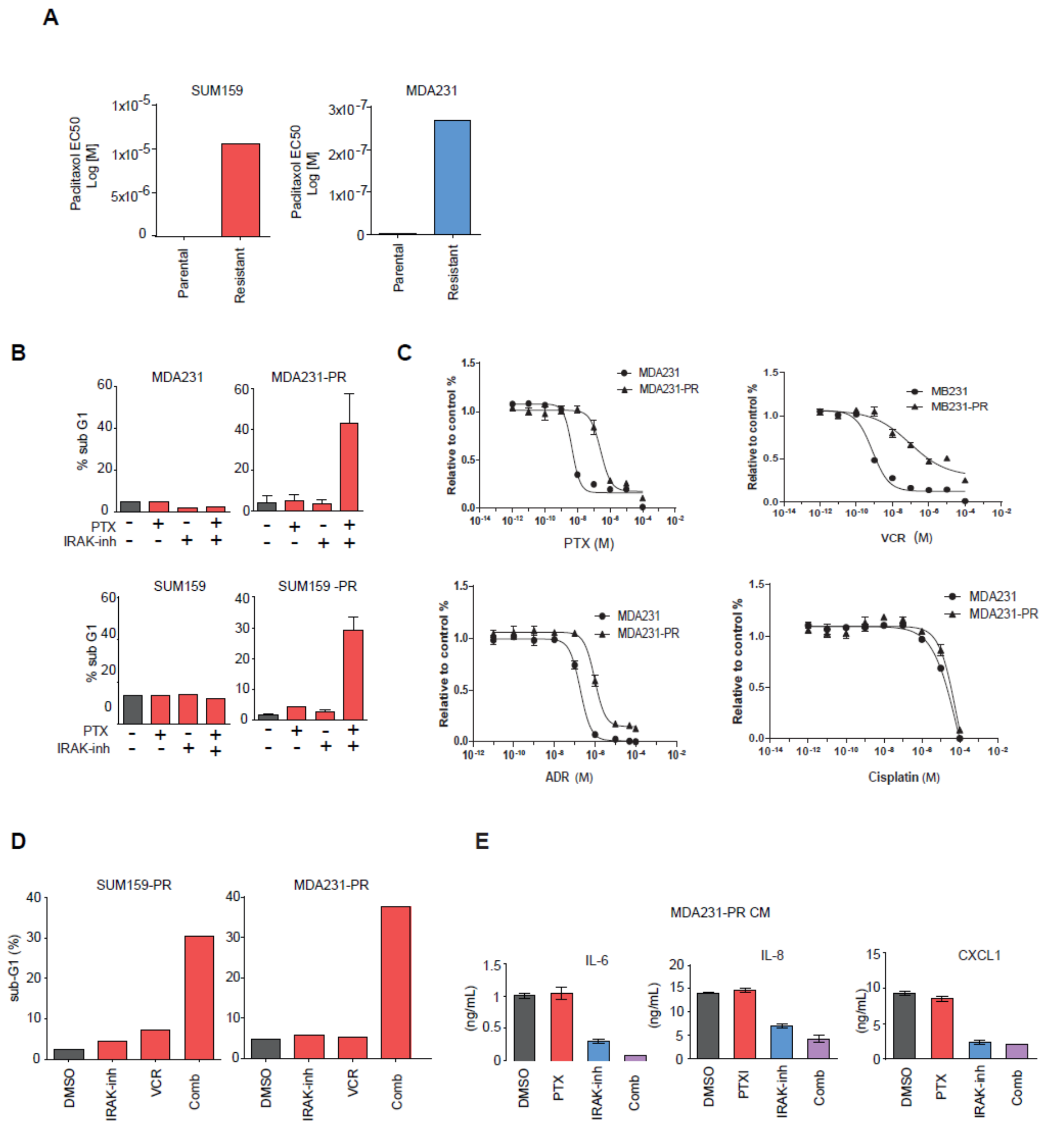

Supplementary Figure 7. IRAK1 signaling in acquired resistance to paclitaxel

(A) Bar graphs showing the EC_{50} of paclitaxel in MDA231 and SUM159 cells parental and paclitaxel resistant lines (PR). **(B) A**poptosis as determined by FACS analysis of cells in Sub-G1, treated with respective doses of PTX, IRAK-inh (5 µM) or both. PTX, 0.43 nM and 75 nM for MDA2131 and MDA231-PR, respectively; 0.73 nM and 1 µM for SUM159 and SUM159-PR, respectively. (**C**) EC50 curves of MDA231 and MDA231-PR cells treated with indicated drugs. (**D**) Apoptosis in PTX resistant cells treated with vincristine (VCR), IRAK-inh (5 µM) or both. VCR, 1µM and 10 nM for SUM159-PR and MDA231-PR, respectively. **(E)** ELISA assay of indicated cytokines in MDA231-PR cells treated with PTX (75 nM), IRAK-inh (5 µM) or both. Error bars represent s.e.m, n=3.

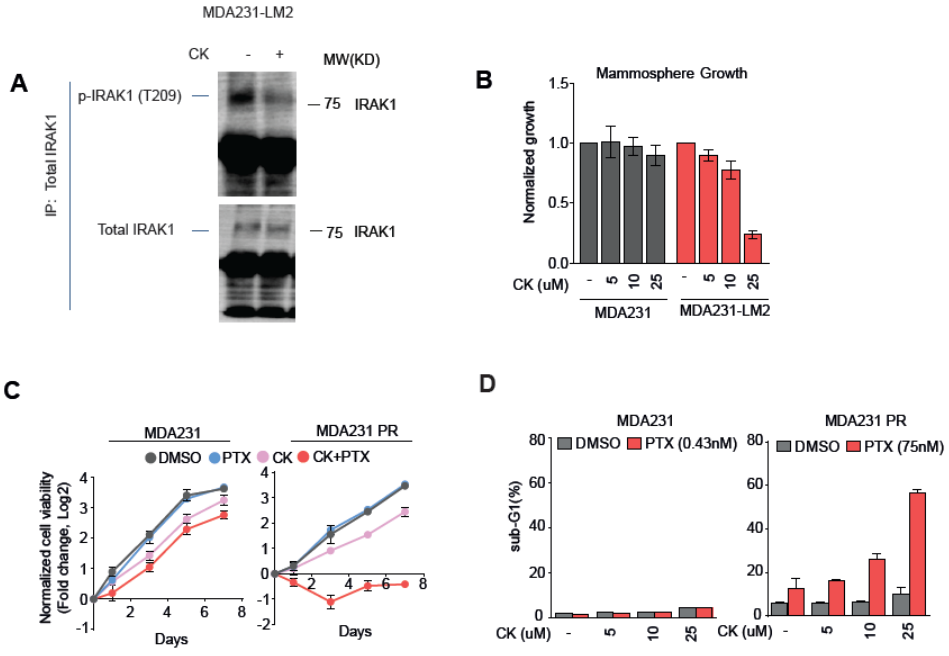

Supplementary Figure 8. Effects of Ginsenoside CK compound on TNBC
(**A**) Western blot showing the effect of CK (25 μM) on p-IRAK1 in MDA231-LM2 cells. (**B**) Effects of CK on mammosphere growth of MDA231 and MDA231-LM2 cells. (**C**) Cell viability assay showing the effect of CK (25 μM) in combination with PTX (0.43 nM and 75 nM, respectively) in MDA231 and MDA231-PR cells. (**D**) Apoptosis via FACS assessment of sub-G1 treated as indicated.

Supplementary Figure 9, Full Western blots

Full unedited gel for Figure 2B

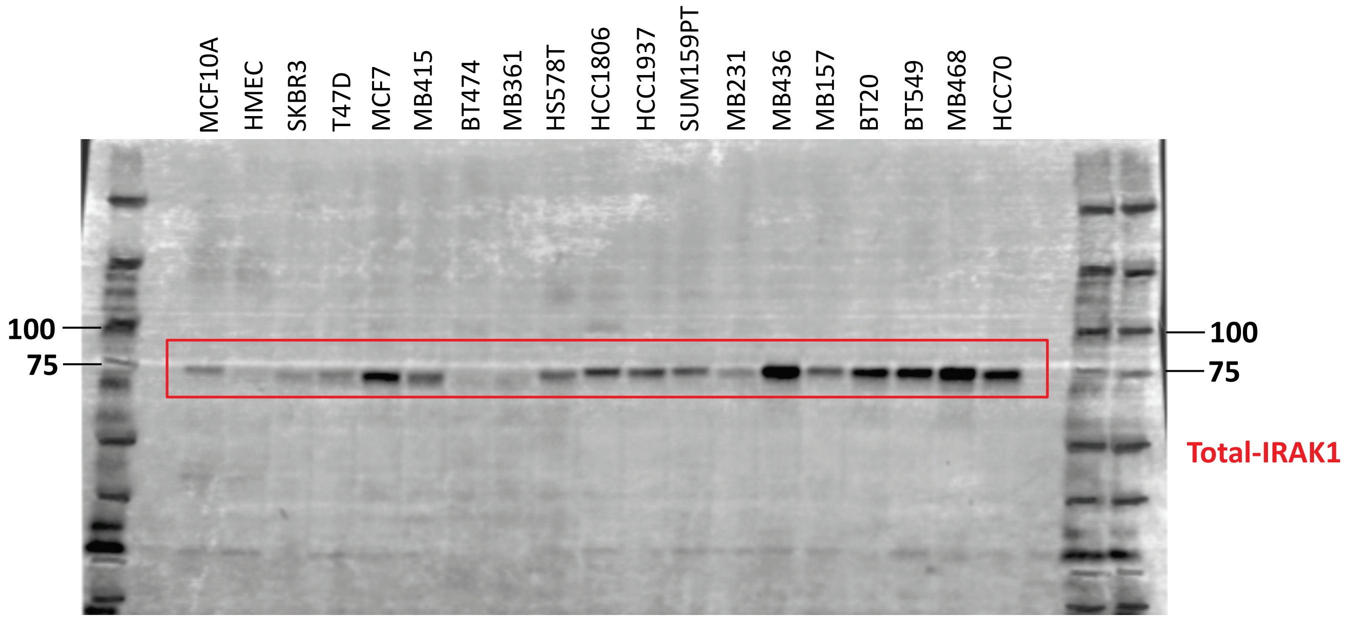

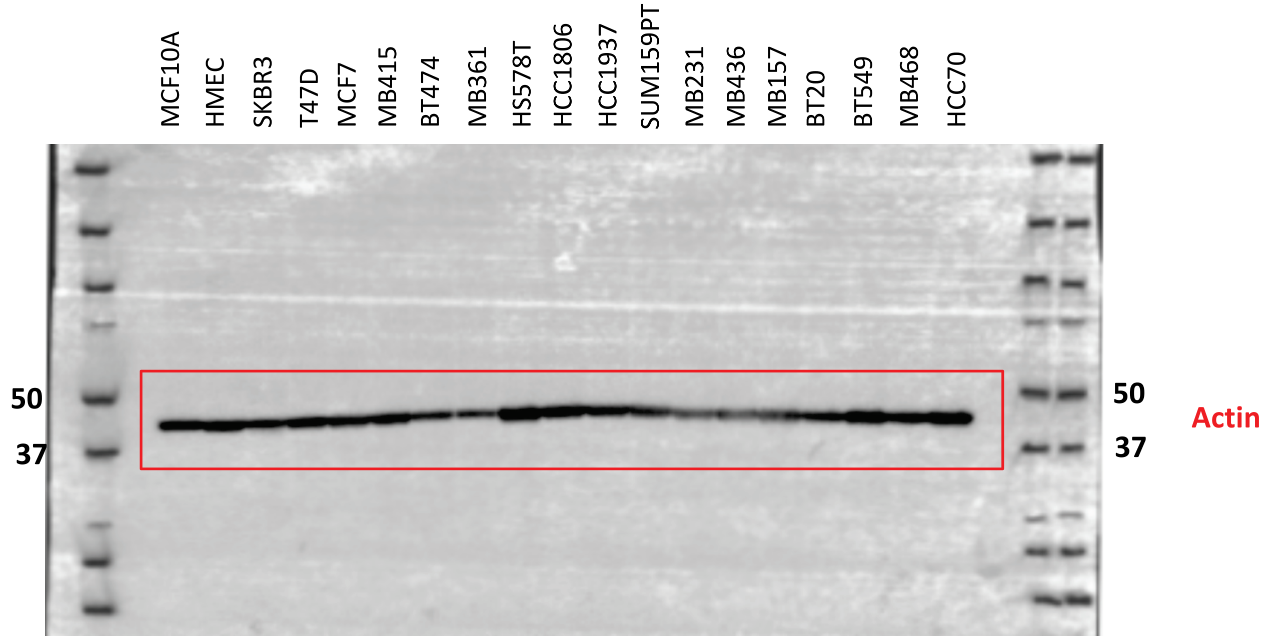

Full unedited gel for Figure 2C

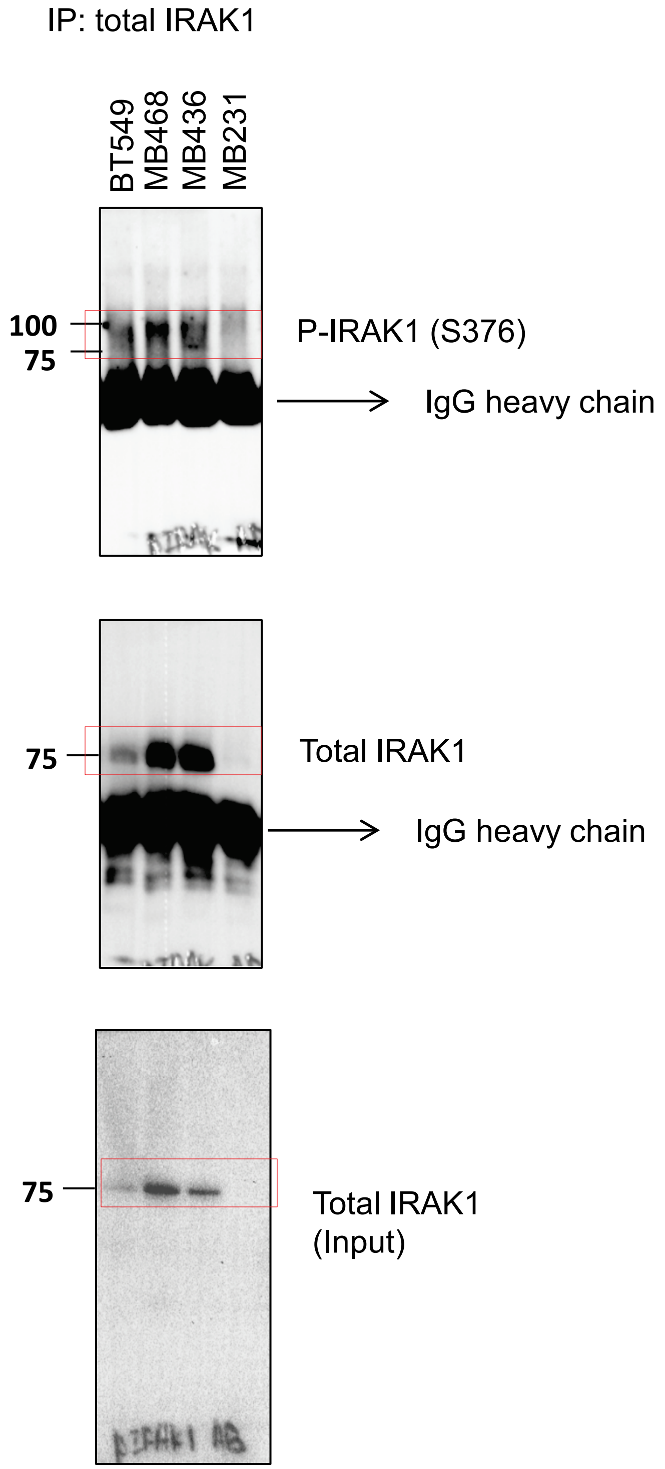

Full unedited gel for Figure 2D

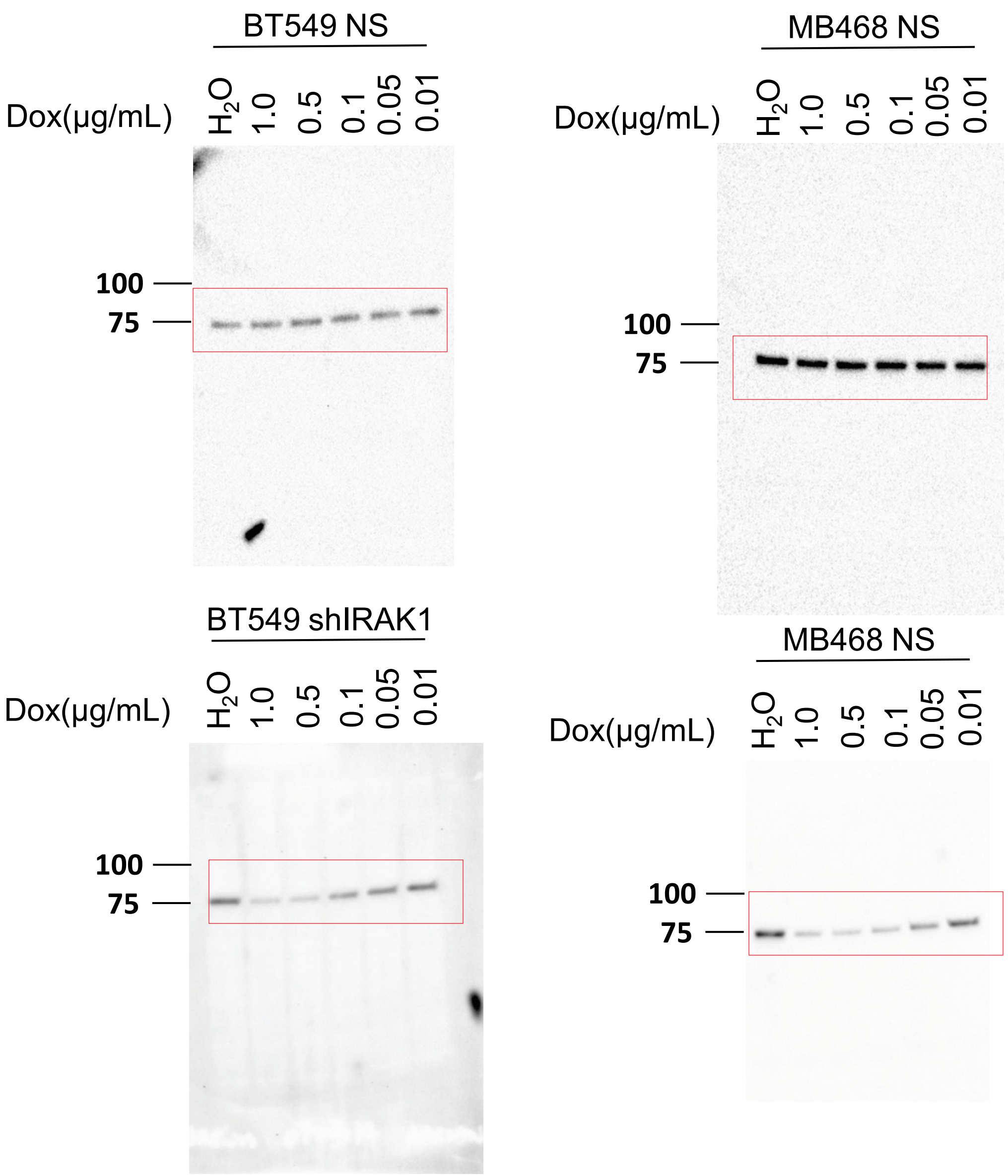

Unedited Western Blots

Full unedited gel for Figure 2H

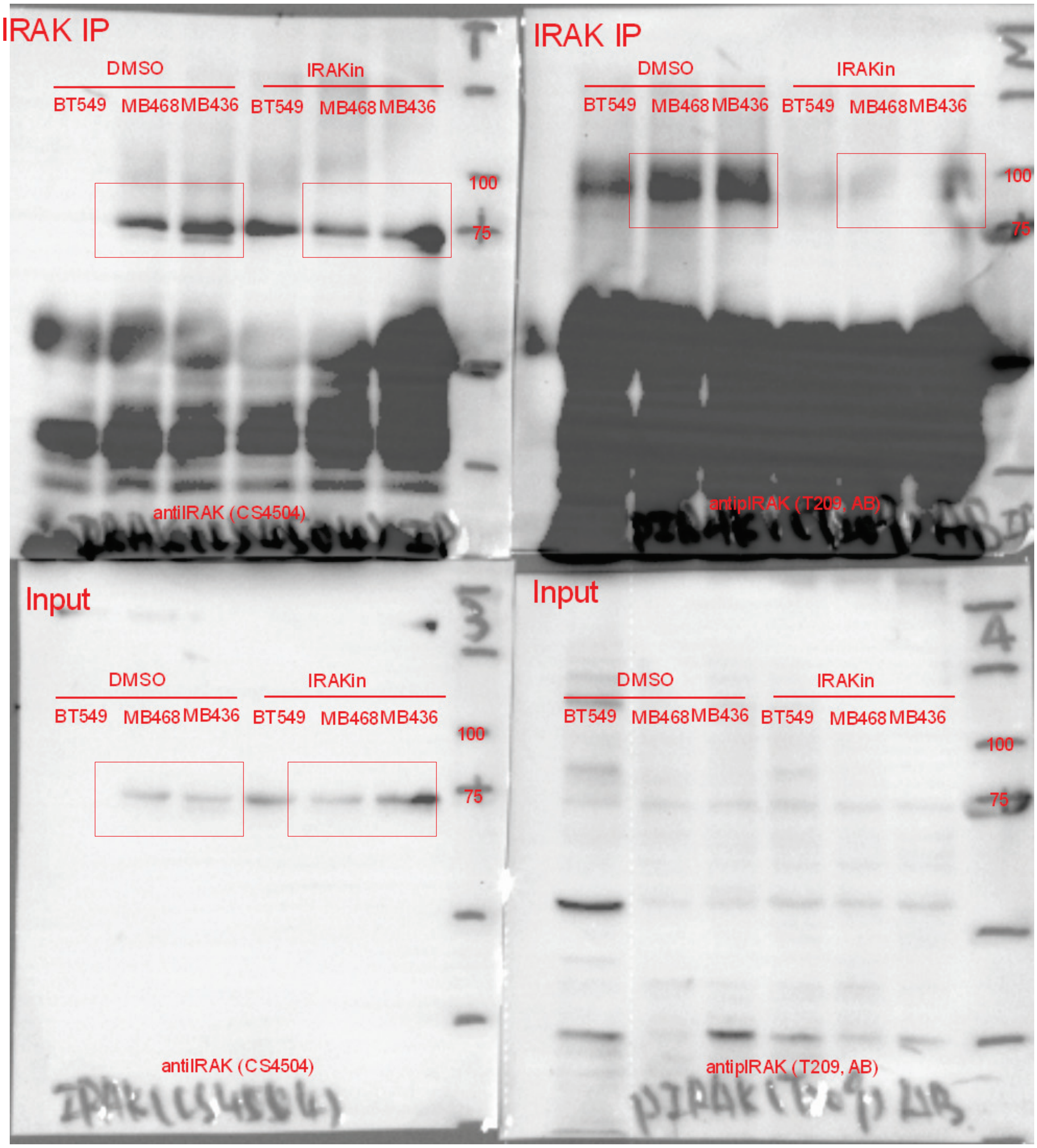

Unedited Western Blots

Full unedited gel for Figure 4D

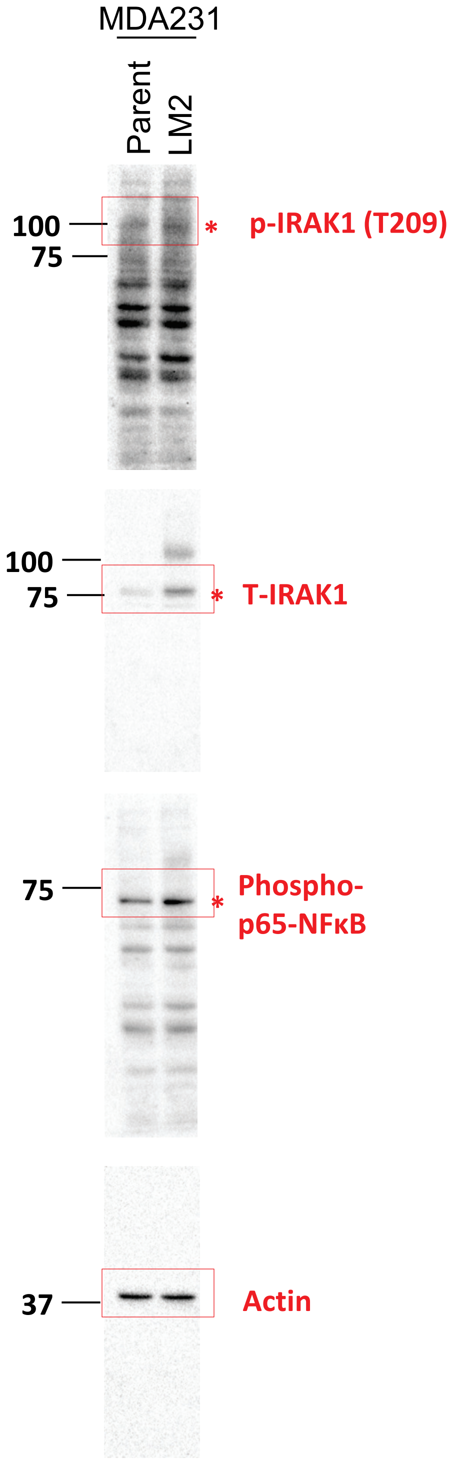

Unedited Western Blots

Full unedited gel for Figure 4E

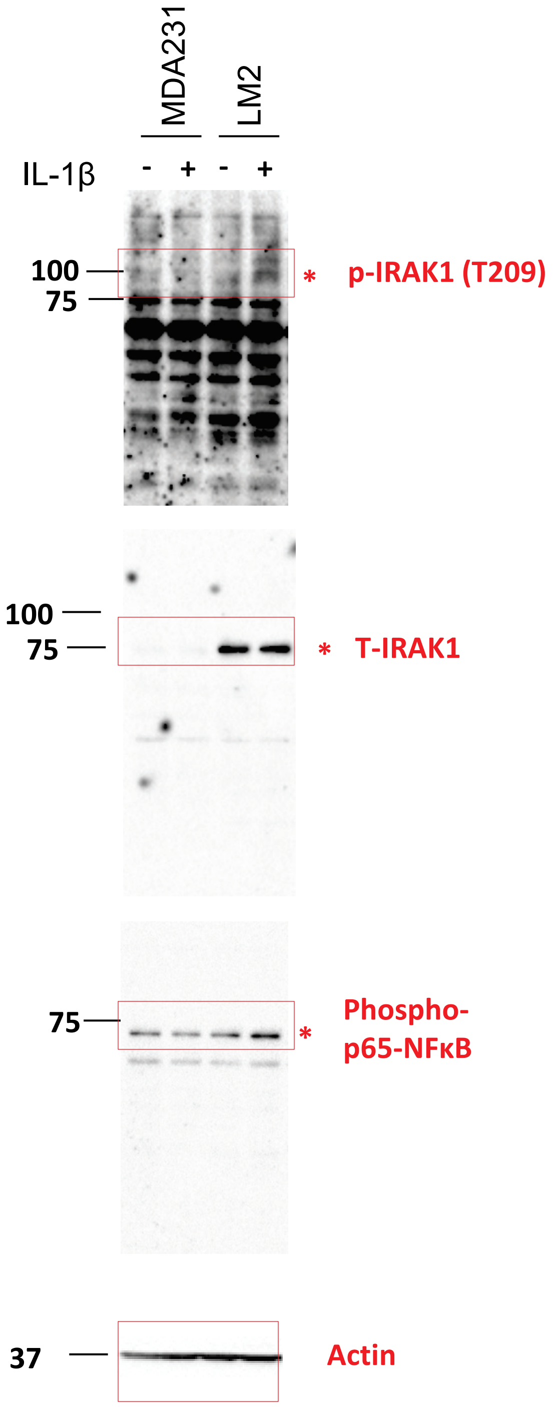

Unedited Western Blots

Full unedited gel for Figure 6A

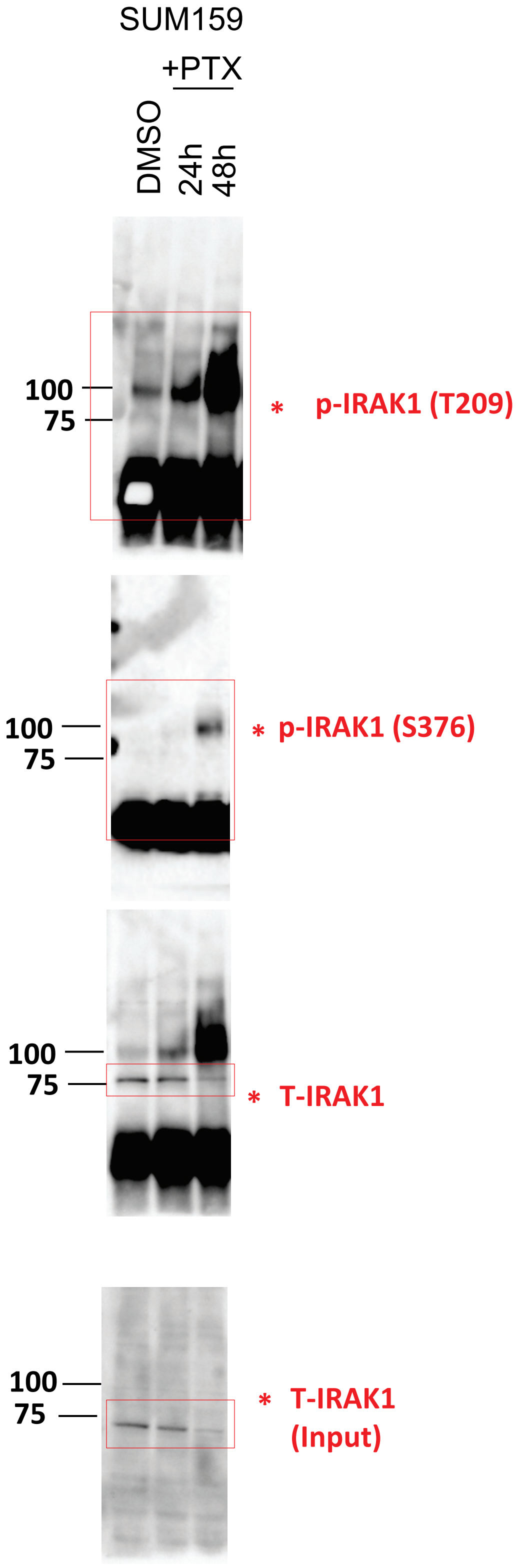

Unedited Western Blots

Full unedited gel for Figure 6E

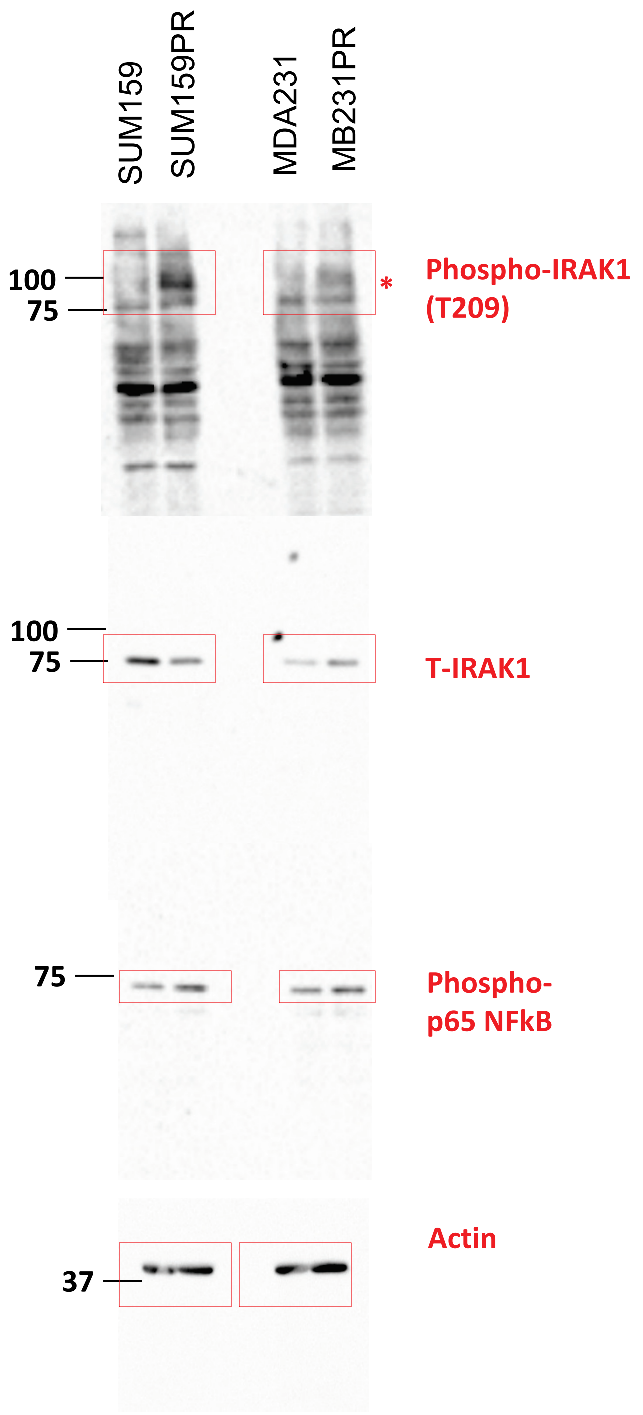

Unedited Western Blots

Full unedited gel for Figure 6H

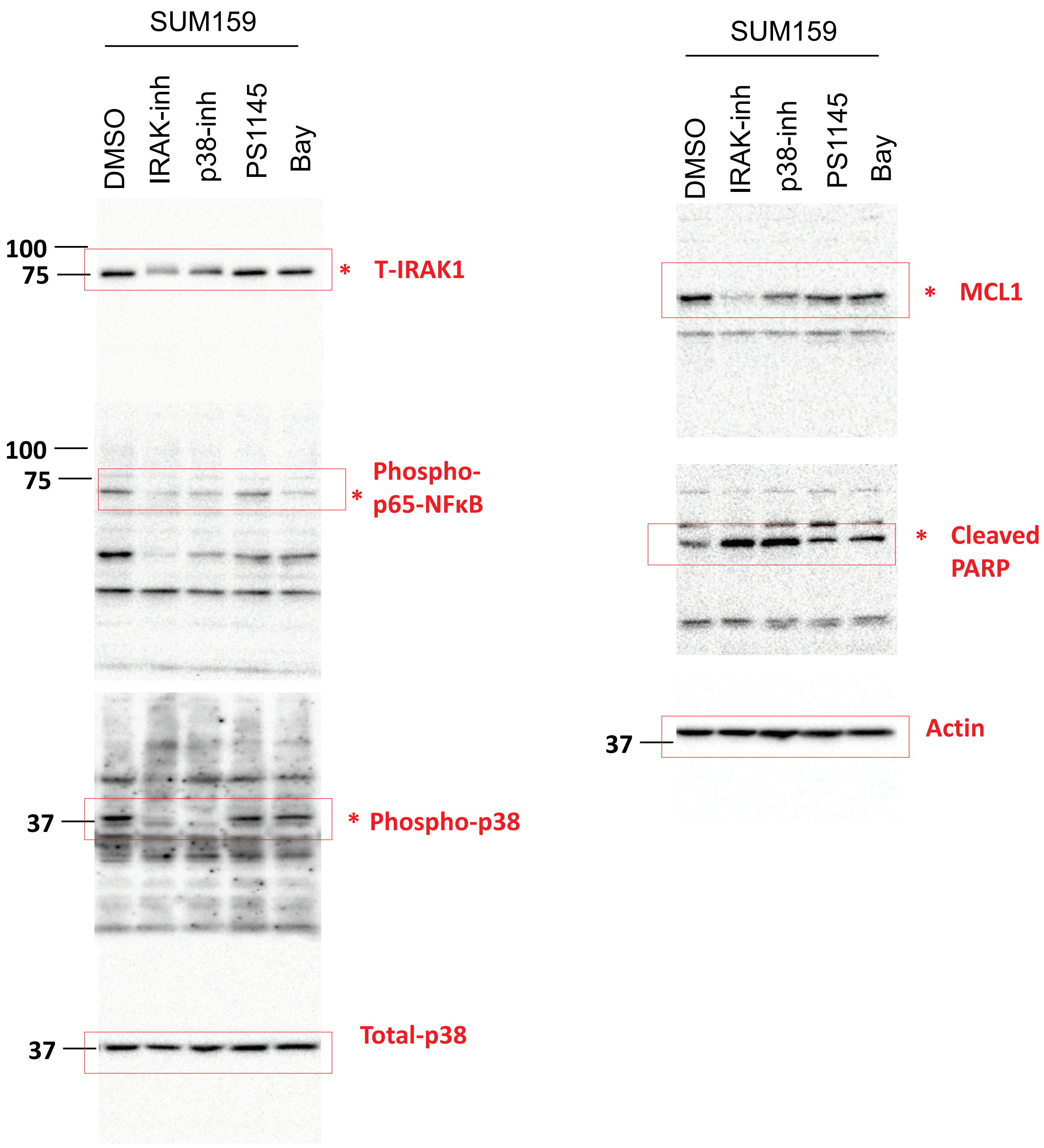

Supplementary Tables

Supplementary Table 1. IRAK1 shRNA constructs and targeting sequences

VECTOR	PLASMID ID	Type of KD	CLONE ID	TARGET	TARGET SEQENCE (5'-3')
pGIPZ	sh1	Constitutive	V3LHS_635467	IRAK1's ORF	AATTCATCACTTTCTTCGG
	sh2	Constitutive	V3LHS_635469	IRAK1's ORF	CCATCACTTTGTAGAAGCG
	shIRAK1 for IRAK1 OE rescue	Constitutive	V3LHS_645859	IRAK1's UTR	ACATGAAACCTGACTTGCT
pTRIPZ	shIRAK1	Inducible	V2THS_132369	IRAK1's UTR	ATTACTCAAGGACAACCTG

Supplementary Table 2. RT-PCR primers

Gene	Forward Primer 5'→3'	Reverse Primer 5'→3'
18S	CGAACGTCTGCCCTATCAACTT	ACCCGTGGTCACCATGGTA
IRAK1	TCAGCTTTGGGGTGGTAGTG	TAGATCTGCATGGCGATGGG
IRAK2	TCTCACCCCCAAACTTGCTC	CCTCGGCCAACACTATTCCA
IRAK3	GCCTGGCAGAGAGACTTTCA	AGGACTCAACACTGCTCCATAG
IRAK4	AGCTTGCAGCAATGGTTGAC	TGTGCCAAGAAAGTGGTGGA
ILIB	GCCAATCTTCATTGCTCAAGTGT	GGTCGGAGATTCGTAGCTGG
IL6	AGTTCCTGCAGAAAAAGGCAAAG	AAAGCTGCGCAGAATGAGAT
IL8	ACCGGAAGGAACCATCTCAC	GGCAAAACTGCACCTTCACAC
CXCL1	CCAGCTCTTCCGCTCCTC	CACGGACGCTCCTGCTG
